# Prediction of gene regulatory enhancers across species reveals evolutionarily conserved sequence properties

**DOI:** 10.1371/journal.pcbi.1006484

**Published:** 2018-10-04

**Authors:** Ling Chen, Alexandra E. Fish, John A. Capra

**Affiliations:** 1 Department of Biological Sciences, Vanderbilt University, Nashville, TN, United States of America; 2 Vanderbilt Genetics Institute, Vanderbilt University, Nashville, TN, United States of America; 3 Departments of Biomedical Informatics and Computer Science, Center for Structural Biology, Vanderbilt University, Nashville, TN, United States of America; Carnegie Mellon University, UNITED STATES

## Abstract

Genomic regions with gene regulatory enhancer activity turnover rapidly across mammals. In contrast, gene expression patterns and transcription factor binding preferences are largely conserved between mammalian species. Based on this conservation, we hypothesized that enhancers active in different mammals would exhibit conserved sequence patterns in spite of their different genomic locations. To investigate this hypothesis, we evaluated the extent to which sequence patterns that are predictive of enhancers in one species are predictive of enhancers in other mammalian species by training and testing two types of machine learning models. We trained support vector machine (SVM) and convolutional neural network (CNN) classifiers to distinguish enhancers defined by histone marks from the genomic background based on DNA sequence patterns in human, macaque, mouse, dog, cow, and opossum. The classifiers accurately identified many adult liver, developing limb, and developing brain enhancers, and the CNNs outperformed the SVMs. Furthermore, classifiers trained in one species and tested in another performed nearly as well as classifiers trained and tested on the same species. We observed similar cross-species conservation when applying the models to human and mouse enhancers validated in transgenic assays. This indicates that many short sequence patterns predictive of enhancers are largely conserved. The sequence patterns most predictive of enhancers in each species matched the binding motifs for a common set of TFs enriched for expression in relevant tissues, supporting the biological relevance of the learned features. Thus, despite the rapid change of active enhancer locations between mammals, cross-species enhancer prediction is often possible. Our results suggest that short sequence patterns encoding enhancer activity have been maintained across more than 180 million years of mammalian evolution.

## Author summary

Alterations in gene expression levels are a driving force of both speciation and complex disease. Therefore, it is of great importance to understand the mechanisms underlying the evolution and function of gene regulatory DNA sequences. Recent studies have revealed that gene expression patterns and transcription factor binding preferences are broadly conserved across diverse animals, but there is extensive turnover in distal gene regulatory regions, called enhancers, between closely related species. We investigate this seeming incongruence by analyzing genome-wide enhancer datasets from six diverse mammalian species. We used two machine learning frameworks (SVMs and CNNs) to train statistical models to distinguish enhancers from the genomic background based on DNA sequence patterns. Both approaches are able to accurately identify enhancers within a species. Furthermore, models trained to predict enhancers from one species can also accurately identify enhancers in the same cellular context in other species—from humans to opossums. This argues that many of the short sequence properties encoding regulatory activity are remarkably conserved across more than 180 million years of mammalian evolution.

## Introduction

Enhancers are genomic regions distal to promoters that bind transcription factors (TFs) to regulate the dynamic spatiotemporal patterns of gene expression required for proper differentiation and development of multi-cellular organisms [1,2]. It is critical to understand the mechanisms underlying enhancer evolution and function, as alterations in their activity influence both speciation and disease [3–5]. Recent genome-wide profiling of TF occupancy and histone modifications associated with enhancer activity revealed that the regulatory landscape changes dramatically between species—both enhancer activity and TF occupancy at orthologous regions distal to promoters are extremely variable across closely related mammals [6–12]. However, the gene regulatory circuits [13] and expression of orthologous genes in similar tissues are largely conserved across mammals [14–16]. Much of the gene regulatory machinery is also conserved; TFs and the short DNA motifs they bind are highly similar between human, mouse, and fly [17–20]. In short, there is considerable change in the enhancer activity of orthologous regions across mammals, despite the relative conservation of gene expression and TF binding preferences.

The rapid turnover in enhancer activity between orthologous regions in different species has largely been attributed to differences in the DNA sequences of the elements involved, rather than differences in the broader nuclear context [21–25]. Genome-wide profiles of TF binding have shown that 60–85% of binding differences in human, mouse, and dog for the TFs CEBPA and HNF4A can be explained by genetic variation that disrupts their binding motifs [23]. Genetic differences are also often responsible for differential enhancer activity between more closely related species; for example, variation in TF motifs at orthologous enhancers was predictive of activity differences between human and chimp neural crest enhancers [25]. This suggests that, while there is turnover at orthologous sequences, sequence properties predictive of enhancer activity may still be conserved.

Until recently, investigation of the conservation of enhancer sequence properties across mammalian evolution has been hampered by a lack of known enhancers across diverse species within the same cellular context. The canonical definition of enhancer activity is the ability to drive expression in transgenic reporter assays [1,26], which cannot currently be scaled to assess regulatory potential genome-wide. However, high-throughput assays such as ChIP-seq can assess histone modifications associated with enhancer activity [27,28] to identify putative enhancers genome-wide in many tissues and species [12,29]. Using known enhancers, machine learning approaches have learned their sequence properties and successfully distinguished enhancers active in specific cellular contexts from both the genomic background and enhancers active in other tissues [30–39]. Moreover, some of these studies suggested the potential for cross-species enhancer prediction. For instance, the similarity of co-occurrence of sequence patterns can be used to identify orthologous enhancers in distantly related Drosophila species [40], and annotated cis-regulatory modules (CRMs) in Drosophila can predict CRMs in highly diverged insect species based on binding site composition similarity [41]. However, TF binding sites have been suggested to evolve and turnover much more rapidly between closely related mammals than Drosophila species [10,42]. Nonetheless, a comprehensive analysis across clades suggests that transcriptional networks and gene regulatory sequences evolve at similar rates across animals [43]. Indeed, in mammals, a machine learning model trained on mouse enhancers accurately predicted orthologous regions of the human genome [31]. However, due to the rapid turnover of enhancer activity between human and mouse, the majority of orthologous regions are not active human enhancers [12]. Overall, these previous studies suggest the potential for evolutionary conservation of sequence properties of mammalian enhancers, but comprehensive genome-wide quantification of the degree and dynamics of this conservation is needed.

In this study, we investigate the degree of regulatory sequence property conservation by applying machine learning classifiers to genome-wide enhancer datasets across diverse mammals. We first confirm that SVM classifiers trained using short DNA sequence patterns can accurately identify many enhancers genome-wide in the adult liver, developing limb and developing brain. Then, by using classifiers trained in one species to predict enhancers in the others, we demonstrate that many enhancer sequence properties are conserved across species, even though the enhancer activity of specific loci is not. We establish the robustness of this conservation to different enhancer identification techniques by showing that classifiers trained using high-confidence human and mouse enhancer sequences validated in transgenic assays also generalize across species and are similar to classifiers trained on histone-modification-defined enhancers. Furthermore, the short DNA patterns most predictive of enhancer activity in each species matched a common set of binding motifs for TFs enriched for expression in relevant tissues. This suggests the patterns learned by classifiers capture biologically relevant sequences that influence TF binding. In addition to SVM classifiers, we also trained CNNs on liver enhancers in each species. The multilayer structures of CNNs are promising for modeling more complex sequence patterns beyond short DNA motifs [36–39,44–46]. The CNNs predicted enhancers with higher accuracy than SVM models, but the CNNs generalized less well across species, suggesting less conservation of some patterns they learned. Together, our results argue that, though there is rapid change of active gene regulatory sequences between mammalian species, many of the short sequence patterns encoding enhancer regulatory activity have been conserved over 180 million years of mammalian evolution. Our findings also suggest avenues for identifying enhancers in species without genome-wide enhancer-associated histone modification data and establish a framework for future exploration of the conservation and divergence of regulatory sequence properties between species.

## Results

### Enhancers can be predicted from short DNA sequence patterns in mammals

Genome-wide enhancer activity across many mammalian species has been assayed via ChIP-seq profiling of enhancer-associated histone modifications in the adult liver [12], developing limb [8] and developing brain [47]. Certain chemical modifications to histones, such as acetylation of lysine 27 of histone H3 (H3K27ac) and lack of trimethylation of lysine 4 of H3 (H3K4me3), are associated with active enhancers and provide a genome-wide proxy for the active enhancer landscape [27,28]. For brevity, we refer to genomic regions with enhancer-associated histone modification combinations identified in these previous studies as “enhancers”.

**Fig 1:**
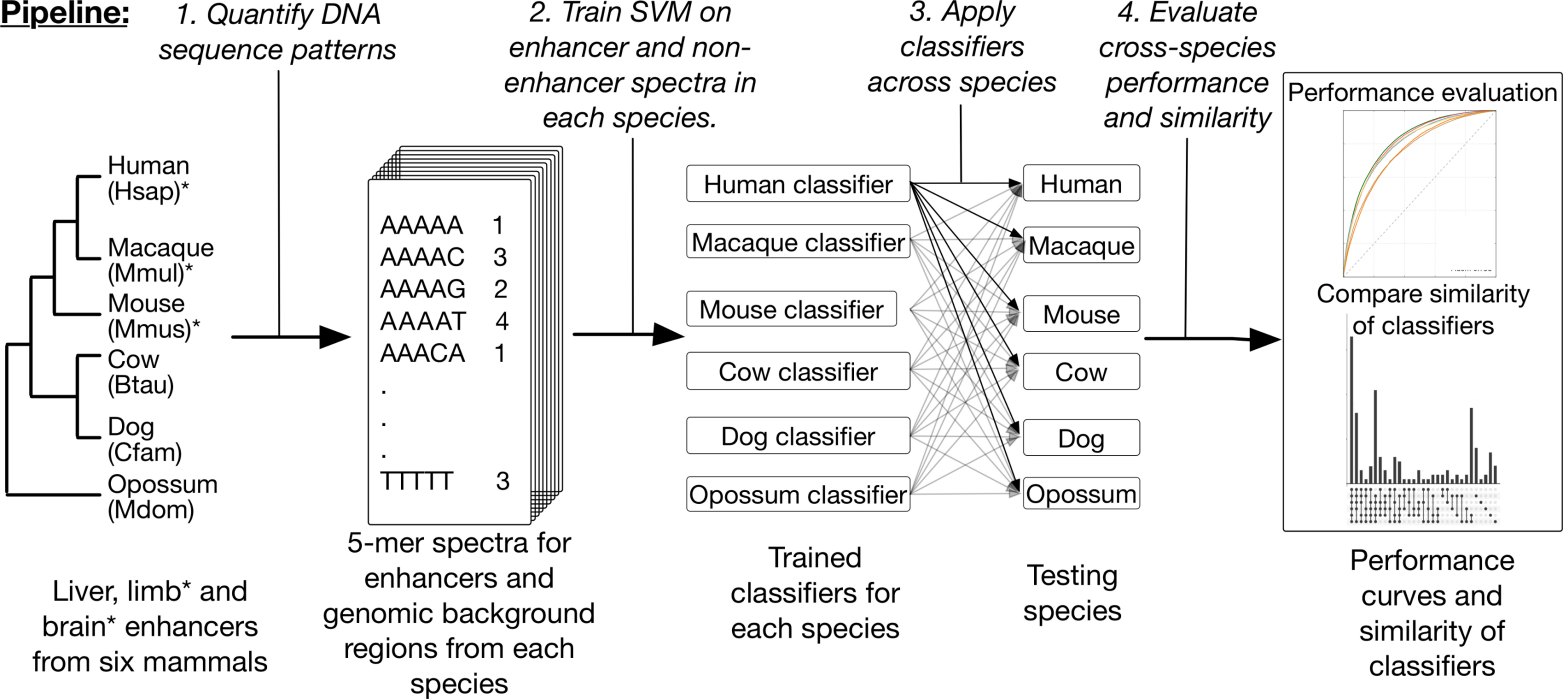
Overview of the framework for evaluating DNA patterns predictive of enhancer activity across diverse mammals. Starting with liver, limb and brain enhancers and genomic background regions from six mammals, the first step of the pipeline quantified each of these genomic regions by their 5-mer spectrum—the frequency of occurrence of all possible length five DNA sequence patterns. Using the spectra as features, we trained a spectrum kernel support vector machine (SVM) to distinguish enhancers from non-enhancers in each species and evaluated their performance with ten-fold cross validation. Then, we applied classifiers trained on one species to predict enhancer activity in all other species. Finally, we evaluated the performance of cross-species prediction compared to within species prediction and quantified the similarity of different species’ classifiers by the sharing of TF motifs among the most predictive 5-mers. Limb and brain enhancer data were only available for human, macaque, and mouse.

For this study, we selected six representative diverse mammals with cross-species enhancer data and high-quality genome builds: human, macaque, mouse, cow, dog, and opossum (Methods). Liver enhancers were available for all species; developing limb and brain enhancers were available for human, macaque, and mouse. For each species and tissue, we evaluated how well short DNA sequence patterns identified enhancers. We trained two machine learning algorithms, *k*-mer SVMs and CNNs, on raw DNA sequence patterns. This approach has the advantage that it is not dependent on previous knowledge of TF motifs. For the *k*-mer SVMs, we quantified DNA sequence patterns present in each genomic region by computing its *k*-mer spectrum—the observed frequencies of all possible nucleotide substrings of length *k*. We then trained SVM classifiers on the *k*-mer spectra to distinguish enhancers from random genomic regions matched to the enhancers on various attributes, such as length, GC-content, and repeat-content, as appropriate. To reflect the fact that most of the genome does not have enhancer activity, we trained and evaluated the SVM classifiers on positive and negative sets containing ten times as many negative non-enhancer regions as enhancers and weighted misclassification costs. We used ten-fold cross validation to evaluate the classifiers, and we quantified performance by computing the average area under receiver operating characteristic (auROC) and precision-recall (auPR) curves over the ten cross-validation folds (Fig 1; Methods). We also trained CNN models for this problem. Due to the challenges of training CNNs, these analyses were performed on balanced training, validation, and testing sets. For all comparisons with SVMs, we compared CNN performance to both the average SVM performance over cross-validation folds and the performance of the SVM on the single CNN test set. (See CNN results and Methods for details.) To document the training setup and performance, we assigned each prediction task an experiment number (S1 Table). We report the experiment number for results throughout the paper for clarity.

**Fig 2:**
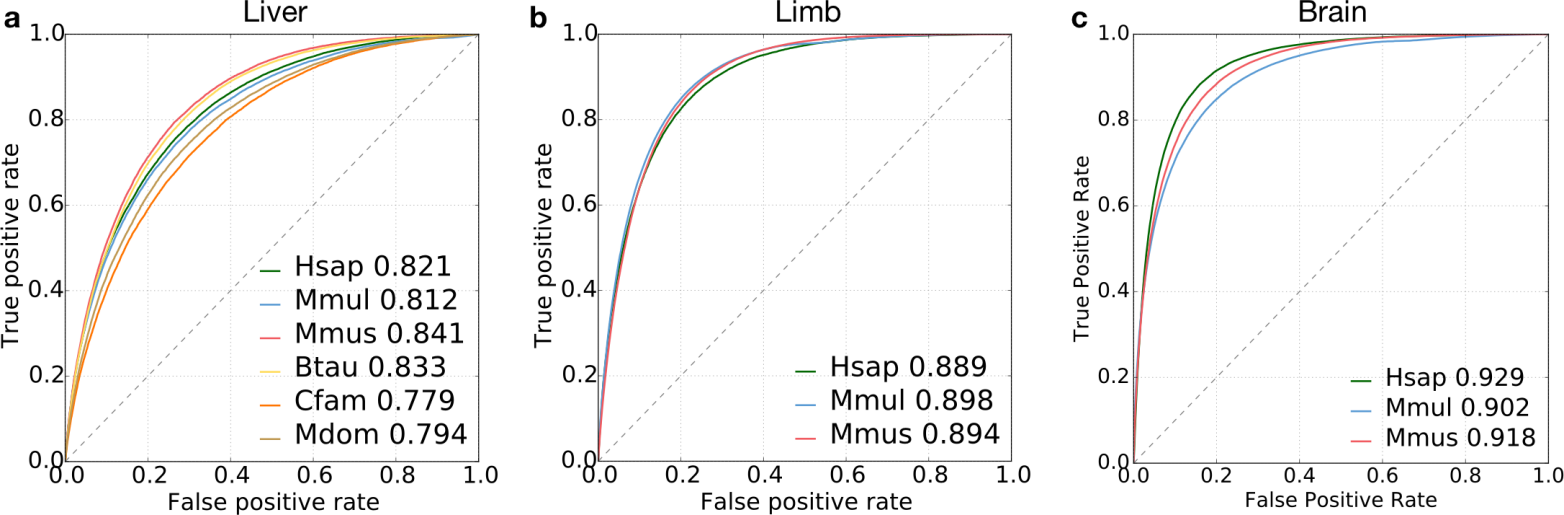
Performance of DNA sequence-based enhancer identification in diverse mammals. (a) ROC curves for classification of liver enhancers vs. the genomic background in six diverse mammals: human (Hsap), macaque (Mmul), mouse (Mmus), cow (Btau), dog (Cfam), and opossum (Mdom). (b) ROC curves for classification of developing limb enhancers in human, macaque, and mouse. (c) ROC curves for classification of developing brain enhancers in human, macaque, and mouse. Area under the curve (AUC) values are given after the species name. Ten-fold cross validation was used to generate all ROC and PR curves (S1a, b, c Fig).

We first evaluated the ability of SVM classifiers trained on 5-mer spectra to identify liver enhancers in the six selected mammals: human, macaque, mouse, cow, dog and opossum (experiments 1, 8, 15, 22, 29, 36). As expected from previous work [31,32,48], all classifiers could distinguish active liver enhancers from length-matched random background regions; auROCs ranged from 0.78 in dog to 0.84 in mouse (Fig 2A; auPRs ranged from 0.27 to 0.35; S1A Fig). Next, we trained 5-mer spectrum SVM classifiers to predict enhancers active in limb (experiments 147, 151, 155) and brain (experiment 165, 169, 173) for human, macaque, and mouse. Again, classifiers accurately distinguished enhancers from the background with even stronger performance than the liver classifiers. The limb classifiers achieved auROCs of ~0.89 in each species (Fig 2B; auPRs from 0.43 to 0.46; S1B Fig), and the brain classifiers had auROCs from 0.90–0.93 (Fig 2C; auPRs from 0.54 to 0.56; S1C Fig). However, we note that the auPRs are lower than auROCs due to the unbalanced training set.

The choice of *k* did not substantially influence performance; the auROCs for human liver classifiers are 0.81, 0.82, 0.82, 0.82, respectively across *k* of 4, 5, 6, and 7. We also explored the application of classifiers based on more flexible *k*-mer features, i.e., the gappy and mismatch *k*-mer kernels (experiments 145, 146) [49], but they did not improve performance (S2 Fig; auROCs of 0.82 and 0.82). The gkm-SVM approach also performed similarly to the *k*-mer SVM on the liver enhancers (auROC 0.76); because of the long computation time of gkm-SVM (experiment 347, Methods), we could only compare it on the balanced liver enhancer set (5-mer SVM auROC of 0.78). These results illustrate that SVMs trained only on DNA sequence patterns can distinguish many enhancers from background sequences across a variety of mammals for three tissues and two developmental time-points.

### Short sequence properties predictive of enhancers are conserved across species

**Fig 3:**
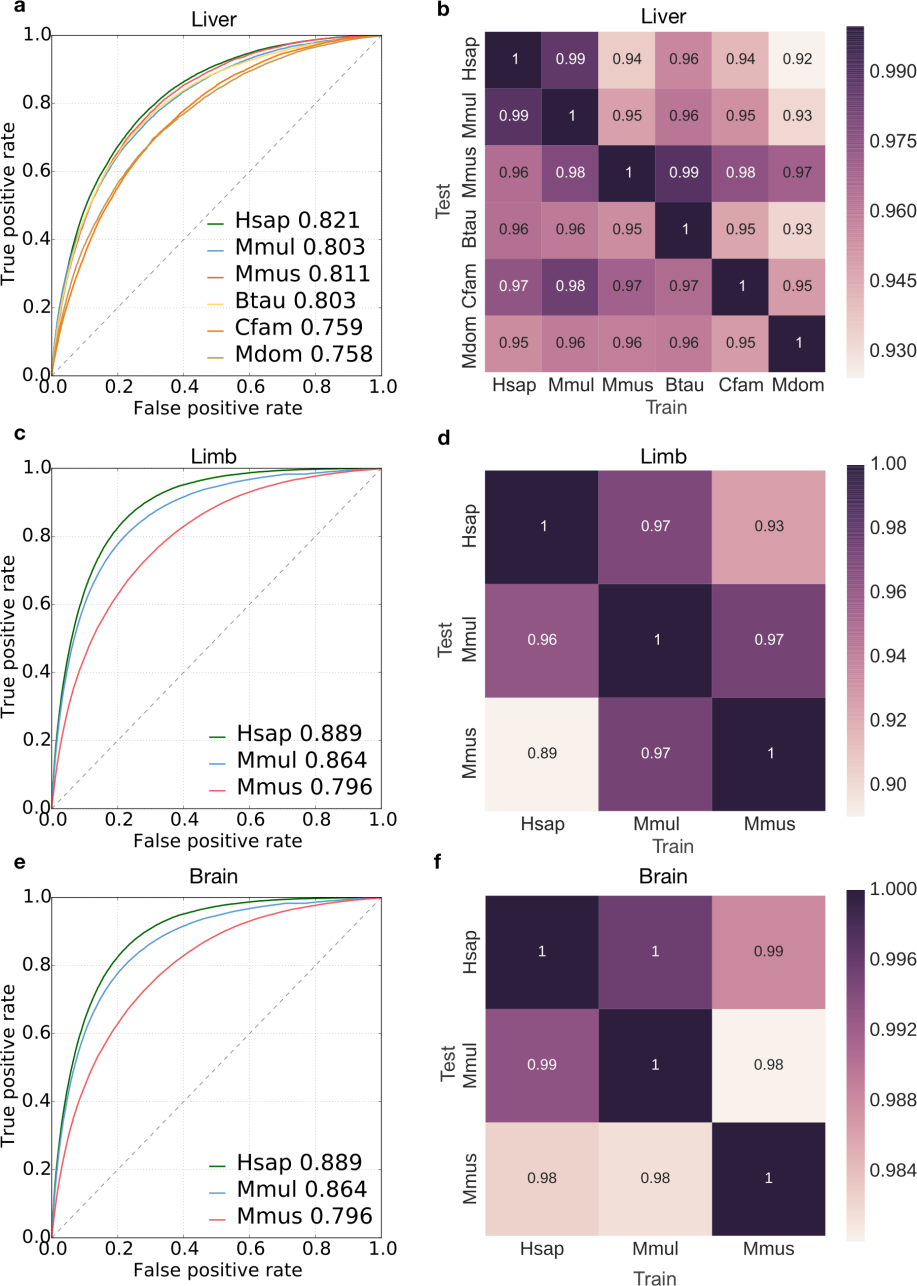
Human-trained enhancer classifiers accurately predicted liver, limb and brain enhancers in diverse mammals. (a) ROC curves of the performance of the human liver enhancer classifier applied to the human (Hsap), macaque (Mmul), mouse (Mmus), cow (Btau), dog (Cfam) and opossum (Mdom) datasets. Area under the curve (auROC) values are given after the species name. (b) Heat map showing the relative auROC of liver enhancer classifiers applied across species compared to the performance of classifiers trained and evaluated on the same species (Fig 2A). The classifiers were trained on the species listed on the x-axis and tested on species on the y-axis. (c) ROC curves showing the performance of the human limb enhancer classifier on human, macaque and mouse. (d) Heat map showing the relative auROC of limb enhancer classifiers applied across species compared to the performance of classifiers trained and evaluated on the same species (Fig 2B). (e) ROC curves showing the performance of the human brain enhancer classifier on human, macaque and mouse. (f) Heat map showing the relative auROC of brain enhancer classifiers applied across species compared to the performance of classifiers trained and evaluated on the same species (Fig 2C). The raw auROC and auPR values for all comparisons are given in S4, S6 and S7 Figs.

We then investigated whether learned DNA sequence patterns predictive of enhancer activity were conserved across mammals by testing whether classifiers trained in one species could distinguish enhancers from the genomic background in another species. First, we applied the human liver classifier to the five other species (experiments 2–6). We quantified cross-species performance using the relative AUCs—the auROC or auPR of the enhancer classifier trained on species A and applied to species B, divided by the average auROC or auPR over cross-validation folds obtained by the classifier trained and tested on species B. In other words, the relative auROC is the proportion of within-species performance achieved by a classifier trained in a different species. The classifier trained on human liver enhancers predicted liver enhancers in other mammals nearly as accurately as classifiers trained in each species (Fig 3A, PR curves in S1D Fig), and its relative performance decreased only slightly across species (Fig 3B, relative auROCs > 95.5%, relative auPRs > 87%). Furthermore, the scores from the human classifier applied to human enhancers were significantly positively correlated with the scores from non-human classifiers (S3 Fig; Spearman’s ρ between 0.90 for macaque and 0.66 for opossum). When expanded to all pair-wise combinations of species (experiments 1–36), classifiers accurately predicted enhancers in every mammalian species tested, regardless of the specific species they were trained in; the average relative auROC was 96.0% (Fig 3B; average relative auPR was 85%, raw AUCs in S4A and S4B Fig). The human classifier was generally the best at cross-species prediction; this is likely due to the higher genome assembly quality and other biases towards human sequences.

Classifiers generalized better to more closely related species; generalization was inversely correlated with the species’ evolutionary divergence, as quantified by substitutions per neutrally evolving site (S5 Fig, Spearman’s rho = –0.4, *P* = 0.14; Methods). This trend became even stronger when controlling for differences in GC content between species (Spearman’s rho = –0.72, *P* < 2.2e–16; S15 Fig).

Classifiers trained to identify enhancers in developing limb and brain also accurately generalized across species. The average relative auROC for the developing limb classifiers was 95.0% across all species pairs (Fig 3C and 3D, S6 Fig), and the average relative auROC for the developing brain classifiers was 98.6% (Fig 3E and 3F; raw AUCs in S7 Fig). The ability of classifiers to generalize to other species illustrates the conservation of sequence properties predictive of enhancers across mammals.

To ensure that the small fraction of liver enhancers shared between pairs of species were not driving performance, we identified human liver enhancers that overlapped enhancers from three other mammalian species with genome-wide multiple sequence alignments (mouse: 13.6%; cow: 20.0%; dog: 16.7%) and vice versa. For each pair of species, the overlapping enhancers were removed from both the human training set and the other species’ testing set, and then new human classifiers were trained and evaluated (experiments 183–188). The classifiers achieved relative auROCs of 0.962 (mouse), 0.957 (cow) and 0.968 (dog), very similar to the analyses that did not remove shared enhancers (mouse: 0.964, cow: 0.964, and dog: 0.974), suggesting that the shared enhancers do not drive the cross-species generalization.

### Enhancers validated in transgenic assays show similar cross-species patterns

Genome-wide mapping of enhancer-associated histone modifications is a cost-effective means to identify putative enhancers; however, the presence of these modifications does not guarantee enhancer activity. Many experimental and computational approaches have been used to identify enhancers [1,50], and there is considerable disagreement among different strategies [51]. To investigate the generality of our conclusions drawn from histone-modification-derived enhancers, we also analyzed enhancers validated *in vivo* via transgenic assays from the VISTA enhancer database. We included six tissues (limb, forebrain, midbrain, hindbrain, heart and branchial arch) with a sufficient number of validated enhancers (> = 50) in human and mouse. Consistent with the results from classifiers trained on histone-modification defined enhancers, the classifiers trained and evaluated on VISTA human enhancers accurately predicted VISTA mouse enhancers in the corresponding tissue from genomic background, and vice versa (experiments 189–212; S8 Fig; average relative auROC = 96.3%, average auPR = 81.6%). This suggests that sequence patterns in enhancers confirmed via reporter assays are conserved between human and mouse. Moreover, the limb classifier trained on H3K27ac regions (excluding VISTA overlaps) accurately predicted VISTA enhancers (auROC = 0.82, auPR = 0.35 in human; experiment 237; Methods) and was competitive with the VISTA-trained limb classifier itself (auROC = 0.80, auPR = 0.39 in human; experiment 238). This suggests that sequence properties predictive of histone-modification defined enhancers are also predictive of enhancers validated in transgenic assays. Thus, in spite of the limited number and biases present in the sequences tested for enhancer activity by VISTA, our models capture conserved sequence attributes of these functionally validated enhancers.

Overall, these results show that the DNA sequence profiles of enhancer sequences captured by species-specific 5-mer spectrum SVM classifiers are predictive of enhancers in other mammalian species in corresponding tissues. The strong generalization of performance and correlation of the predictions for specific sequences by classifiers trained in different species indicates that many sequence properties predictive of enhancers are conserved across mammals.

### Short DNA sequence patterns remain predictive of enhancer activity after controlling for GC content and repetitive elements

**Fig 4:**
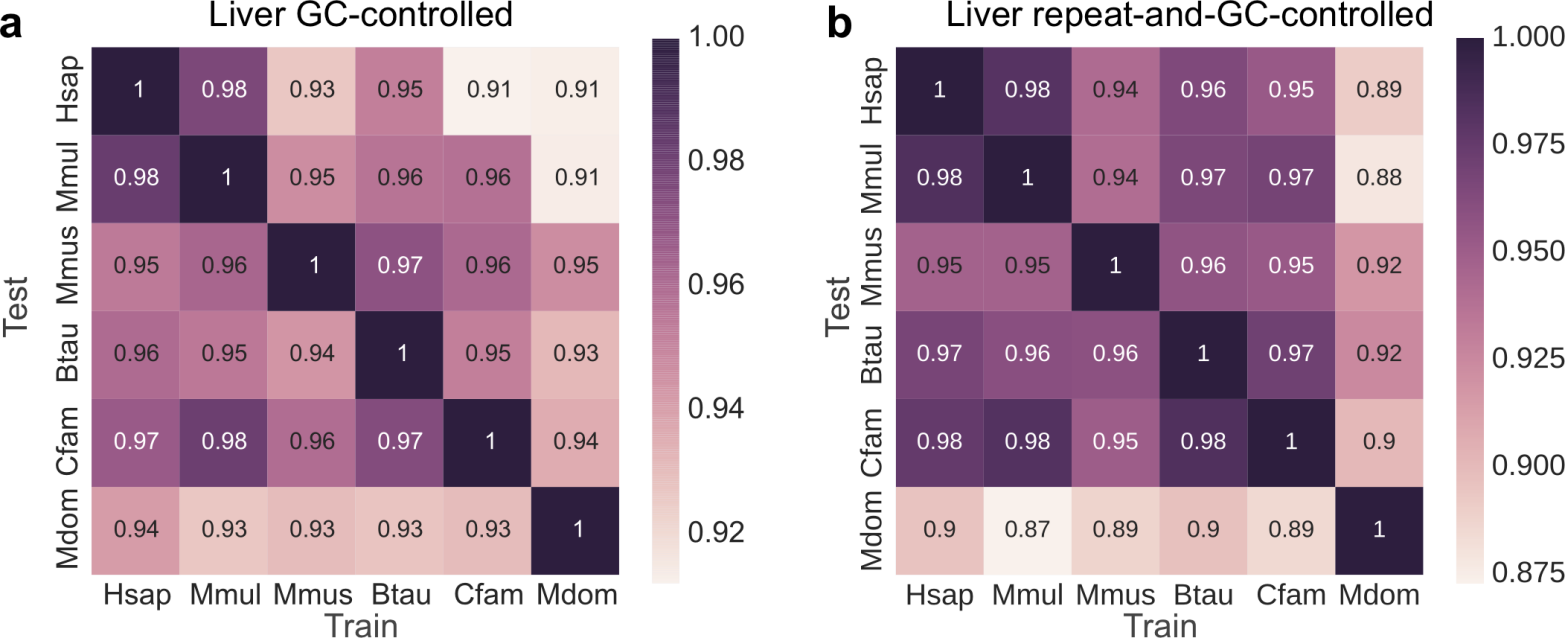
Enhancer sequence properties remain conserved across diverse mammals after controlling for both GC-content and repetitive elements. The heat maps give the cross-species relative auROCs for SVM classifiers trained on 5-mer spectra to identify enhancers in the species along the x-axis, and then used to predict enhancers in the species on the y-axis. The “negative” training regions from the genomic background were matched to the enhancers’: (a) GC-content, and (b) GC-content and proportion overlap with repetitive elements.

Enhancer activity is positively correlated with GC content (S3G Fig), and enhancers are often born from repetitive sequences derived from transposable elements [52–55]. Thus, we sought to evaluate the extent to which these properties influenced the generalization of our enhancer prediction models across species. First, we trained GC-controlled classifiers using negative sets of random genomic regions matched on GC content (experiments 37–72, 156–164, and 174–182). The predictive power of the GC-controlled classifiers was substantial (average auROC of 0.75 for liver, 0.79 for limb and 0.81 for brain; average auPR of 0.24 for liver, 0.28 for limb and 0.34 for brain; S9–S11 Figs, S1 Table), but as expected, less than the corresponding classifiers without GC-control (average auROC of 0.81 for liver, 0.89 for limb and 0.92 for brain; Fig 2). Nevertheless, GC-controlled classifiers maintained strong cross-species generalization: liver classifiers had an average relative auROC of 94.8% (average relative auPR of 86.3%) when applied to the other five species (Fig 4A); limb classifiers had an average relative auROC of 95.0% (relative auPR of 82.4%) when applied across species (S10E Fig); brain classifier had an average relative auROC of 94.8% (relative auPR of 84.2%, S11E Fig). We observed similar cross-species generalization with classifiers trained on VISTA enhancers and GC-controlled negatives as well (experiment 214–237, S12 Fig). The enhancer predictions for individual sequences by the GC-controlled classifiers were significantly correlated, and as expected, high GC-content sequences no longer received consistently high scores (S13 Fig). Ultimately, the strong cross-species generalization of the GC-controlled classifiers suggests that enhancers differ from the genomic background in sequence patterns beyond GC-content, and that those patterns are conserved. In addition, we trained classifiers to distinguish enhancers from their flanking regions (within 10x enhancer length) (experiments 372–407). These classifiers also performed similarly and generalized across species (S14 Fig); this suggests that the conserved sequence properties are specific to enhancers, not just regulatory genomic neighborhoods.

The generalization of each liver GC-controlled classifier across species had the same pattern as the classifiers without GC-control: the human classifier had the best generalization (average relative auROC = 96.1%), while the opossum had the worst (average relative auROC = 92.8%), which is likely due to the quality of genome assembly. In these GC-controlled analyses, we observed a stronger inverse correlation between the relative performance across species and sequence divergence (S15 Fig, Spearman’s *rho* = –0.72, *P* < 2.2e–16) than in the non-GC-controlled analysis (S4 Fig). This indicates that both genomic differences in GC content distribution and overall evolutionary divergence influence the conservation of the sequence patterns predictive of putative enhancers.

To evaluate the influence of repetitive elements on the ability to distinguish enhancers from the background and the observed conservation of sequence properties across species, we trained classifiers to distinguish enhancers that did not overlap a repetitive element (only 3.3% of all enhancers in human) from matched non-repetitive regions from the genomic background (experiments 73–108). Neither the ability to distinguish enhancers from the background in a species, nor the ability of predictive sequence properties to generalize across species, was substantially reduced (S16 Fig). This demonstrates that, while repetitive elements contribute to enhancer activity, the conservation of sequence properties predictive of enhancers is not contingent on their presence.

To further examine the influence of GC-content and repetitive elements across all observed enhancer sequences, we also trained classifiers to distinguish all enhancer regions from genomic background regions matched for both GC-content and the proportion of overlap with a repeat element (experiments 109–144). The performance of these classifiers slightly decreased (average auROC of 0.73, auPR of 0.21; S17 Fig) relative to when not controlling for repeat overlap (average auROC of 0.75, auPR of 0.24; S9A Fig) or neither repeats or GC-content (average auROC of 0.81, auPR of 0.32; Fig 2). This indicates that, as expected, both features are informative about enhancer function. However, the repeat and GC-controlled classifiers still generalized across species (average relative auROC = 94.0%, Fig 4B; average relative auPR = 85.4%); this demonstrates that enhancer sequence properties beyond both GC and repeat content are conserved across species.

#### Enhancer sequence properties are more similar across the same tissue in different species than across different tissues in the same species

**Fig 5:**
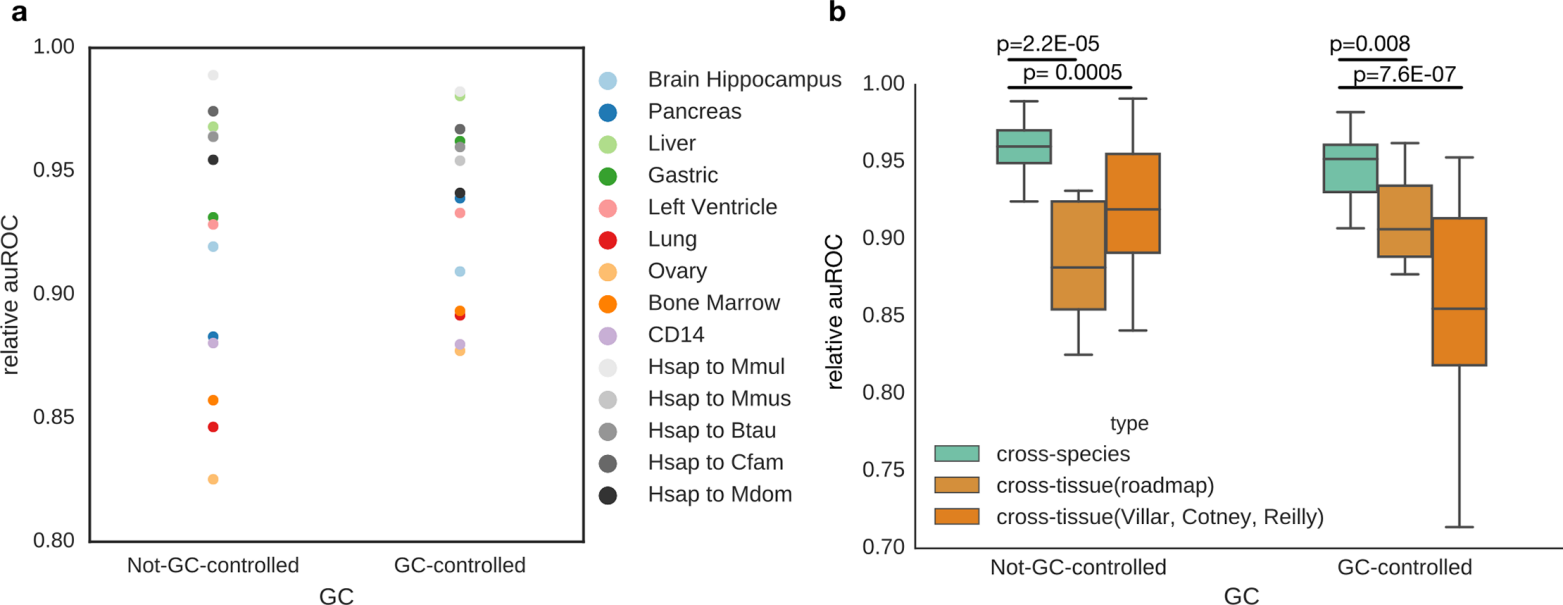
Enhancer classifiers generalize more accurately across the same tissue in different species than across different tissues in the same species. (a) The human-trained liver classifier obtains better performance when applied to liver enhancers from other species (gray dots) than when applied to enhancers from other human tissues. This also holds for GC-controlled analyses, with the exception of predicting enhancers active in the gastric mucosa. (b) In the not-GC-controlled analysis, the cross-species performance (average relative auROC = 96.2%) is significantly better than the cross-tissue (roadmap) performance (88.4%, Mann Whitney U test, *P* = 0.00005) and the cross-tissue (Villar, Cotney, Reilly) performance (92.0%, Mann Whitney U test, *P* = 2.2E-05). This also holds true for the GC-controlled analysis. The cross-species performance (average relative auROC = 94.6%) is significantly better than the cross-tissue (roadmap) performance (91.2%, Mann Whitney U test, *P* = 0.008) and the cross-tissue (Villar, Cotney, Reilly) performance (85.8%, Mann Whitney U test, *P* = 7.6E-07).

Gene expression patterns are significantly more similar in corresponding tissues across species than between different tissues in the same species [14–16], and we demonstrated that enhancer sequence properties are strongly conserved in the same tissue across species (Fig 2). Thus, we hypothesized that, as for gene expression, enhancer sequence properties would be more similar in the same tissue across species (cross-species) than between different tissues in the same species (cross-tissue). To test this, we performed cross-tissue analysis using human enhancers identified in nine diverse cellular contexts, including liver, by the Roadmap Epigenomics Project [2] (experiments 239–274; Methods). We applied the classifier trained on human liver enhancers (from Villar et al. 2015) to Roadmap Epigenomics enhancers from: liver, brain hippocampus middle, pancreas, gastric, left ventricle, lung, ovary, CD14 cells, and bone marrow. We compared the relative auROC between the cross-tissue and cross-species prediction tasks (Fig 5A). In the non-GC-controlled analysis, the human liver enhancer classifier predicts enhancers in macaque, mouse, cow, dog and opossum better than all non-liver Roadmap tissues. In the GC-controlled analysis, we observed the same trend. The cross-species predictions are more accurate than cross-tissue predictions, with the exception of the Roadmap gastric tissue (dark green), which is also a digestive tissue. When compared to the relative auROCs of all pairwise cross-species analyses in liver, limb and brain, those of human liver to non-liver Roadmap tissues are significantly lower (all *P* < 0.008, Mann-Whitney U test; Fig 5B). In addition to the human cross-tissue analysis, we also examined the cross-tissue performance of the liver, limb and brain classifiers over all three species with enhancer data: human, macaque and mouse. For each species, we applied the classifiers trained in liver, limb, and brain to that species’ enhancers in the other two tissues. Again, cross-species performance (all pairwise relative auROCs) was significantly higher than cross-tissue performance in both GC-controlled and non-GC-controlled analyses (Fig 5B). We observed the same trend in relative auPRs (S18 Fig). The ability of enhancers to regulate gene expression is often contingent on both cell-type specific attributes, such as expression patterns of TFs [56], and properties that are shared across active enhancers in general. The stronger performance of the trained classifiers in the cross-species compared to cross-tissue prediction tasks suggests that they capture cell-type-specific sequence attributes and that these features are conserved across species.

### The most predictive sequence patterns in different species match binding motifs for many of the same transcription factors

**Fig 6:**
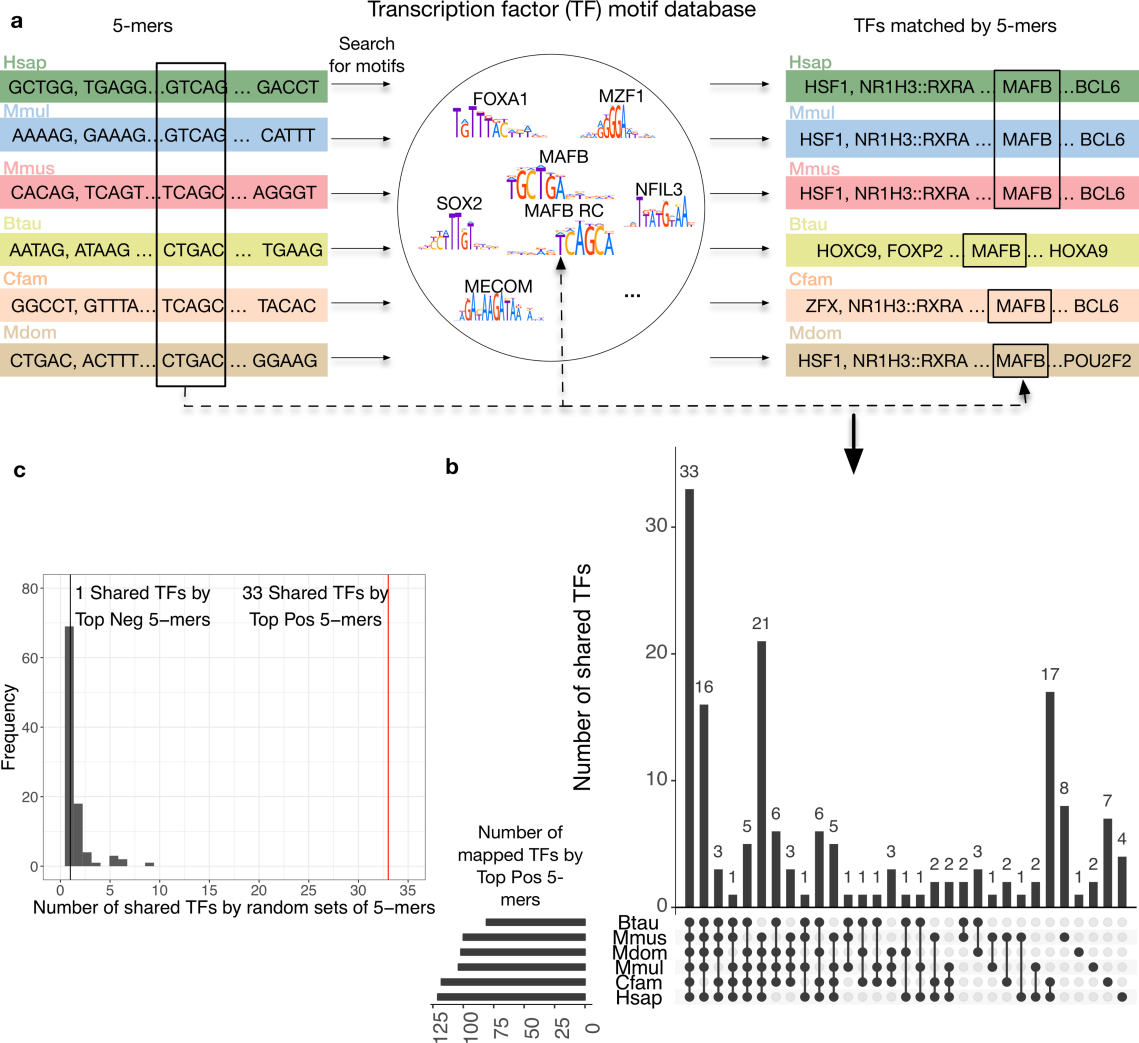
The DNA sequence patterns most predictive of liver activity across species matched a common set of transcription factors. (a) Transcription factor analysis workflow. For each species enhancer classifier, we found TF motifs matched by the top 5% positively weighted 5-mers. Note that different 5-mers (marked with black box on the left) can match the same motif, e.g., MAFB and its reverse complement (RC). The overlap of matched TFs were then compared across each species’ classifier. (b) 33 of the TF motifs matched by the top 5% positive 5-mers from each GC-controlled liver classifier are shared in all species. The total number of TFs matched by top 5-mers in each species was: 121 (human), 104 (macaque), 100 (mouse), 81 (cow), 118 (dog), 102 (opossum). Similar results were observed for the non-GC-controlled classifier (S20 Fig). (c) The number of TFs matched by all species based on 5-mers in top positive, top negative, and 100 random sets of 5% of all possible 5-mers. The 33 TF motifs shared among the high-weight set for each species is thus significantly more than expected.

To interpret the biological relevance of the sequence patterns learned by the trained SVM enhancer prediction models in each species, we analyzed the similarity of the sequence properties in a functional context: TF binding motifs. First, we matched the 5% (n = 52) most enhancer-associated 5-mers learned by the human GC-controlled liver classifier to a database of 205 known TF motifs [57] using TOMTOM (Fig 6A). The enhancer-associated 5-mers were significantly more likely to match at least one TF motif than expected at random (46.1% vs. 27.7%; one-tailed *P* = 0.0035, binomial test). The 5% (n = 52) most background-associated 5-mers were not significantly different from random (21.6% matched at least one TF, two-tailed *P* = 0.43, binomial test). This illustrates that the classifiers learned sequence patterns with regulatory potential.

Next, we investigated whether the TF binding motifs matched by enhancer-associated 5-mers were shared between species. The highly weighted 5-mers in the human-trained classifier matched 121 TF motifs. Of these, the binding motifs for 33 TF were also matched by enhancer-associated 5-mers in all other species (Fig 6B, S1 Table). This is significant enrichment for shared TF motifs among the enhancer-associated 5-mers compared to the number of TF motifs shared across all species on average over 100 random sets of 5% of 5-mers from each species (Fig 6C, *P* = 0). Similarly, only one TF motif (MZF1) was shared among all the species’ most background-associated 5-mers; this is not significantly different from the number expected at random (*P* = 0.97). Moreover, the sharing of TFs matched by the top positive 5-mers between the human liver classifier and other species’ liver classifiers is significantly higher than that between the human liver classifier and classifiers for other human tissues (*P* = 0.019, Mann Whitney *U* test; S19 Fig). This also suggests more conservation of enhancer sequence properties across species within the same tissue than within the same species across different tissues. We obtained similar results when comparing the TFs matched by 5-mers from non-GC-controlled liver SVM models (27 shared TFs by enhancer-associated 5-mers in all species, *P* = 0, S3 Table, S20 Fig). The limb and brain classifiers also shared more TFs among the top 5% of enhancer-associated 5-mers than expected from random sets: 12 TFs (*P* = 0.33, GC-controlled) and 20 TFs (*P* = 0.1) were shared among the limb classifiers; 22 TFs (*P* = 0.05, GC-controlled) and 16 TFs (*P* = 0.14, non-GC-controlled) were shared among the brain classifiers (S3 Table). However, it is likely that the smaller number of available species for developing limb and brain enhancers, our limited knowledge of binding motifs for TFs active in developing limb and brain, and the heterogeneity of developing limb and brain tissue reduced power to detect sharing compared to liver.

To evaluate the relevance of the shared TF motifs to liver function, we analyzed expression patterns of the TFs across 12 tissues [58]. Shared TFs among liver enhancer-associated 5-mers were significantly enriched for liver expression (Table 1, *P* = 0.011, one-tailed Fisher’s exact test), and 6 out of the 7 shared TFs not expressed in liver have a liver-expressed TF in the same subfamily (S2 Table). Many of the shared TFs play essential roles in liver function. For instance, they are enriched for activity in the TGF-β signaling pathway compared to non-shared TFs; the enrichment is mainly due to members of the AP-1 (JUN, FOS, and MAF subfamilies) and SMAD families (Methods) [59,60]. TGF-4 signaling is a central regulatory mechanism that is disrupted in all stages of chronic liver disease [61]. Further, mice deficient in c-JUN or MAF have an embryonic lethal liver phenotype [62,63]. The only TF shared among the background-associated 5-mers, MZF1, is lowly expressed in the liver and not detected at the protein level [64]. We also searched for matches to the binding motifs of known liver master regulators among the highly weighted motifs. While none of them were shared among all models, several including, HNF1A, HNF4A, and FOXA1 matched highly weighted motifs in three or more species (S3 Table). This demonstrates that the sequence patterns learned in each species capture similar motifs that are recognized by TFs important to the relevant tissue context.

**Table 1:** The TFs with motifs shared among the top 5-mers across all species’ liver enhancer SVM classifiers are significantly enriched for liver expression (*P* = 0.011, one-tailed Fisher’s exact test).

	*Shared TFs*	*Not shared TFs*
*Liver expressed*	26	89
*Not liver expressed*	7	70
*Percent Liver expressed*	78.8%	56.0%

### Convolutional neural networks predict enhancers more accurately than SVMs, but generalize less well across species

**Fig 7:**
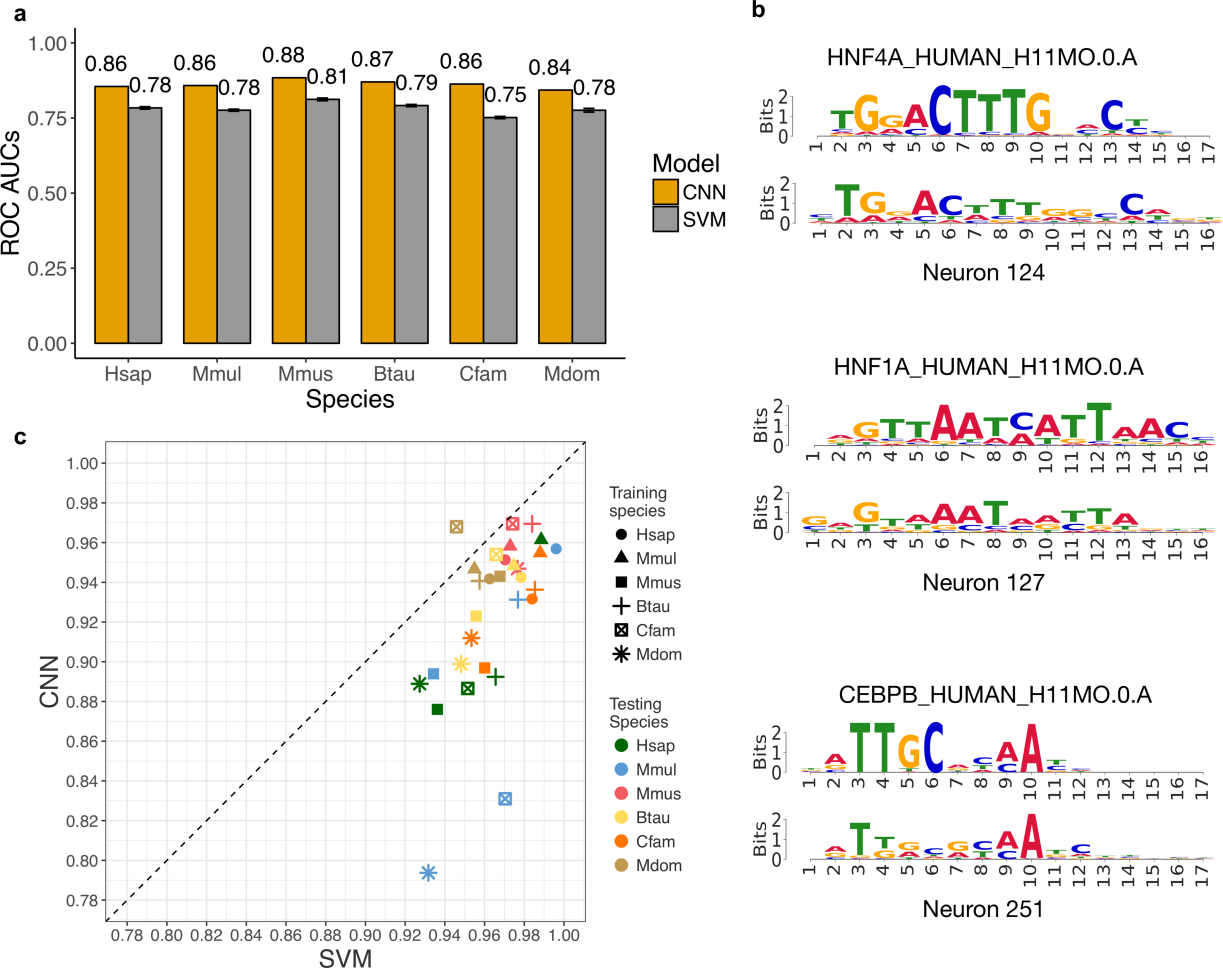
CNNs identify enhancers more accurately than 5-mer spectrum SVM models, but generalize less well across species. (a) The auROCs of CNN models are substantially better than the 5-mer SVM model in each species. The error bars give the standard error of ten-fold cross-validation for the SVM models. (b) Neurons in the first layer of the CNN learned the motifs of important liver TFs, including HNF4A, HNF1A, and CEBPB. (c) The relative auROCs of the CNN models applied across species are consistently lower than for the 5-mer SVMs applied across the same species. This suggests that the CNN models do not generalize as well across species as the SVM models.

Convolutional neural networks have recently achieved the state-of-art performance at predicting regulatory sequences [36,37] and may be better at capturing more complex sequence patterns than *k*-mer SVMs. To investigate the performance and generalization of CNNs at identifying enhancers across species, we trained CNNs to distinguish liver enhancers from the random genomic background in each species. Here, we used the center 3000 bp of enhancers and a balanced negative set due to the fixed-length input of CNNs and the challenges of training CNNs on unbalanced sets (Methods). To compare the performance of CNNs with the SVM models, we trained a CNN model (experiment 275), a 5-mer spectrum SVM classifier (experiment 325), a 5-mer polynomial kernel SVM (Methods, experiment 351) and an 11-mer gkm-SVM (Methods, experiment 347) on the same human dataset using training, validation, and testing partitions to avoid overfitting (Methods). We found that the k-mer SVM, polynomial kernel SVM, 11-mer gkm-SVM achieved similar performance on this human dataset, with auROCs of 0.78, 0.78, 0.76 and auPRs of 0.75, 0.76, 0.76, respectively (S21 Fig). The CNN performed considerably better, achieving an auROC of 0.86 and auPR of 0.84. Although we did not explore the full hyperparameter space for SVMs, CNN out-performed SVMs by a substantial margin. This was true for the three SVM algorithms we tested across a range of Cs (S21 Fig). Because the prohibitive runtime of gkm-SVM (Methods) and small difference in performance, we continued the CNN comparison with the 5-mer spectrum SVMs. We trained CNNs (experiment 275–310) and 5-mer spectrum SVMs (experiment 311–346) on the 3,000 bp long, balanced enhancer datasets for each species and performed cross-species enhancer predictions (S22 Fig). The CNN model is substantially better than the SVMs at distinguishing enhancers from genomic background in each species (Fig 7A), suggesting that the ability to model more complex sequence patterns improves predictions. Moreover, the first layer of the human liver CNN learned many binding motifs for TFs relevant to liver biology, including CEBPB, HNF4A, and HNF1A (Fig 7B).

Next, we performed the cross-species enhancer prediction with the CNNs. The CNN models generalize well across species (relative auROC from 0.79 to 0.97), but their generalization is consistently worse than the SVM models (Fig 7C; Raw auROCs and auPRs is in S22A and S22C Fig). We observed similar results with repeat and GC-control, and the removal of shared orthologous enhancers (S23 Fig). In addition, we applied the 5-mer polynomial kernel SVM across species to test if the worse generalization of the CNNs could be explained by its ability to capture k-mer interactions (experiments 408–443). The polynomial kernel SVMs perform similarly to the k-mer SVMs within species and do not generalize substantially worse than k-mer SVMs across species, suggesting little influence of global k-mer interaction patterns on enhancer identification (S24 Fig). This is consistent with the finding that the co-binding patterns of TFs are mostly conserved between human and mouse [65]. These results suggest that the sequence properties learned by the CNNs are less conserved across species than those learned by the k-mer spectrum SVMs. These could include better representations of TF motifs or more sophisticated interactions between TFs, such as their orientation, spacing and ordering. However, developing clear biological interpretations for the patterns learned by the CNNs is challenging.

## Discussion

In this study, we trained SVM and CNN classifiers based on DNA sequence patterns to distinguish enhancers from the genomic background in diverse mammalian species. We showed that, in spite of significant changes in the enhancer landscape between species, the SVM models trained using short sequence patterns as features exhibited minimal decreases in performance when applied across species. This indicates that short sequence patterns predictive of enhancer activity captured by these models are largely conserved across mammals. Furthermore, the DNA patterns most predictive of liver enhancer activity across species matched a common set of TF binding motifs with enrichment for expression in the relevant tissue. The sequence properties predictive of histone-mark defined enhancers were also predictive of enhancers confirmed in transgenic reporter assays. We then showed that CNN models performed better than SVMs at identifying enhancers. They also generalized well across species, but not as well as SVMs. These results suggest that conserved regulatory mechanisms have maintained constraints on short sequence motifs present in enhancers for more than 180 million years.

Confidently identifying and experimentally validating enhancers remains challenging [51]. We showed that short sequence properties are conserved across species using enhancers identified via two complementary techniques: histone modification profiling and transgenic assays. Each of these approaches has strengths and weaknesses. The histone-modification based enhancer predictions enable genome-wide characterizations across many species, but this approach is prone to false positives [66,67]. On the other hand, the transgenic assays clearly demonstrate the competence of a sequence to drive gene expression, but are restricted to a biased set of relatively few sequences from two species that are tested at one developmental stage. By showing the cross-species conservation is maintained in both categories, and that models trained on each set perform similarly, we argue the conservation of enhancer short sequence properties is robust to the methodology used to define enhancers.

The design of this study can serve as a framework for further examining the conservation and divergence of regulatory sequence patterns across species. We trained sequence-based machine learning models within a species, and then applied them to other species; this approach can be applied on a genome-wide scale, is not dependent on knowledge of TF binding motifs, and allows some flexibility in the weights assigned to each feature while directly testing the generalization of overall sequence patterns. Identification of enhancers in more divergent species would enable us to better quantify the depth of enhancer sequence properties conservation. This remains an open question, as more divergent animal species have very little conservation of TF co-associations at putative enhancers despite conservation of TF binding preferences [68]; however, enhancer properties appear to be conserved over greater evolutionary timescales in insects [41,42,69] and transcriptional networks seem to evolve at relatively constant rates across animals [43]. Identification of enhancers in the same cellular context for more closely related species would also enable the investigation of lineage-specific regulatory sequence patterns. Thus, additional comparative studies of regulatory sequence features in more species are needed to better understand both recent and ancient influences on regulatory sequences.

While both the SVM and CNN classifiers correctly distinguished many enhancers from the genomic background, neither performed perfectly. Many factors contribute to this, including: false positives in the training data, noise from the low resolution of the histone modification peaks (i.e., they include non-functional sequence flanking the enhancer), errors in the genome assemblies, and the features considered in our models. As enhancer datasets and prediction methods improve, it will be valuable to continue to evaluate generalization across species. It will also be valuable to train, evaluate, and interpret CNNs on unbalanced data sets. Additionally, the features learned by the enhancer CNNs are difficult to interpret biologically, especially for higher-level neurons. Thus, it is not clear whether the CNN classifiers achieved better performance within species but had worse generalization across species by capturing sophisticated interactions between simpler motifs, by more accurately modeling the sequence-preferences of TFs in each species, via better recognition of the genomic background, or recognition of other unappreciated patterns. The interpretation of sequence features learned by the accurate CNNs could facilitate the understanding of how more complex rules of enhancer sequence architectures change during evolution. The identification and interpretation of conserved and diverged gene regulatory patterns between species is an important area for future work.

### Conclusions

We demonstrated that short DNA sequence patterns predictive of enhancer activity learned in one species generalize very well to other mammals. This suggests evolutionary conservation of the short sequence motifs in enhancer sequence architectures between mammals. The commonality of short sequence elements predictive of enhancer activity argues that much of what we learn about enhancer biology, particularly at the basic sequence motif level, in model organisms could be extrapolated to humans. Sequence-based cross-species enhancer prediction could be of particular use in studying difficult to obtain human tissues and providing preliminary annotations in uncharacterized species and tissues. There is also the potential to combine sequence-based models with successful cross-species enhancer prediction strategies based on functional genomics data [70]. Furthermore, our framework could also be adapted to investigate conservation of other functionally relevant factors, such as histone modifications and DNA shape [10,71]. Nonetheless, much work remains to understand how regulatory programs are robust to sequence changes, yet receptive to functional divergence, and to facilitate our interpretation of the effects of non-coding variants in diverse mammals.

## Methods

### Genomic data

All work presented in this paper is based on hg19, rheMac2, mm10 (mouse liver dataset), mm9 (mouse limb and brain dataset), bosTau6, canFam3 and monDom5 DNA sequence data from the UCSC Genome Browser. For consistency with the original studies, liver gene annotations are from Ensembl v73, limb and brain gene annotations are from Ensembl v67 [72]. The sequence divergence between each pair of species was computed from the tree model built from fourfold degenerate sites in the 100-way multiple species alignment from UCSC Genome Browser (http://hgdownload.cse.ucsc.edu/goldenpath/hg19/phastCons100way/hg19.100way.phastCons.mod).

### Enhancer and genomic background datasets

We evaluated the ability of machine learning models to distinguish different sets of enhancers (positives) from sets of matched regions from the genomic background (negatives). In this section, we describe the collection and processing of the enhancer and genomic background sets. In the next section, we describe the training and evaluation of the SVM classifiers.

We analyzed three multi-species histone-modification-defined enhancer datasets in this study. The first consisted of liver enhancers identified by genome-wide ChIP-seq profiling of histone modifications (H3K27ac without H3K4me3) in 20 species from five mammalian orders [12]. These regions are almost entirely distal to coding regions (i.e., more than 1kb away from the nearest TSS) [73]. We use the definition of “high-quality genomes” from Villar et al. 2015 [74]. We selected a member of each order with a high-quality genome build for analysis when possible; however, the most diverged order—marsupials—did not have a species with a high-quality genome build. We consequently selected opossum, as it was the most diverged from humans. For all analyses, we did not consider enhancers or random regions that fell in genome assembly gaps (UCSC gap track) when generating negatives. For human and mouse, we also excluded the ENCODE blacklist regions [58] (https://sites.google.com/site/anshulkundaje/projects/blacklists). This resulted in the following number of observed enhancers in each species: human (N = 29152), macaque (N = 22911), mouse (N = 18517), cow (N = 30892), dog (N = 18966), and opossum (N = 23160) [12]. A small fraction of liver enhancers overlapped with one another (3.0% in human, 2.0% in macaque, 3.0% in mouse, 6.2% in cow and 1.6% in dog), and the overlaps were mostly under 10% of the enhancers’ lengths. Thus, these overlaps are unlikely to cause overfitting during within-species cross-validation runs. We also performed cross-species analyses both with and without orthologous sequences.

We generated four different sets of matched genomic background regions for use as negatives in the training and evaluation of the liver classifiers for each of the six species. The first are random genomic regions matched on length and chromosome to the observed enhancers. Second, for the GC-controlled analyses, we generated genomic background regions matched to the enhancers on length, chromosome, and GC-content. Third, for the repeat controlled analysis, we obtained repetitive elements identified by RepeatMasker for each species [75] and generated random regions from the genomic background matched on length, chromosome, GC-content, and proportion overlap with repetitive elements. Finally, we generated negatives using flanking regions of enhancers. We define the flanking region of an enhancer is 10 times of its length on either side. We then randomly select 10 negative regions of same length as the enhancer that do not overlap other enhancers from the candidate flanking regions. To reflect the fact that enhancers make up a small portion of the genome, we chose an imbalanced data design with 10 times as many of the genomic background (negative) regions as there were enhancers.

The second enhancer dataset contained human (N = 25304), macaque (N = 88560), and mouse (N = 87406) enhancers identified from profiling the H3K27ac modification in developing limb tissue [8]. The third enhancer dataset contained human (N = 48853), macaque (N = 57446), and mouse (N = 51888) enhancers identified from profiling the H3K27ac modification in developing brain tissue [76]. For limb and brain enhancers, we excluded regions within 1 kb of a transcription start site. For each species, we combined the enhancer regions from different development stages. The genomic background regions for each species were defined following the same procedure as for the liver enhancers.

To determine how well classifiers generalized across additional tissue types, we used human enhancers identified by the Roadmap Epigenomics Project [2] in nine tissues from diverse body systems: liver (GI, E066), hippocampus middle (brain, E071), pancreas (exocrine-endocrine, E098), gastric (GI, E094), left ventricle (heart, E095), lung (E096), ovary (reproductive, E097), bone marrow derived mesenchymal stem cell cultured cells (stromal-connective, E026) and CD14 primary cells (white blood, E029). We defined enhancers in these tissues as H3K27ac without H3K4me3 regions. For each tissue, we generated not-GC-controlled and GC-controlled negative training examples as described for the liver enhancers above.

In addition to the histone-modification-defined enhancers, we also analyzed enhancers validated in transgenic reporter assays in embryonic day 11.5 mouse embryos from VISTA [77]. We investigated all six tissues with at least 50 positive enhancer elements in both species: forebrain, midbrain, hindbrain, limb, heart and branchial arch. These enhancers comprised the positive training examples. For each positive, we generated 10 length and chromosome matched random genomic regions as negative training examples. There are not enough failed reporter assays across all selected tissues to generate ten sets of negatives, and there are biases in how the human and mouse regions were selected for testing in VISTA. Thus, we did not use classifiers trained on regions with failed reporter assays as negatives for cross-species analyses.

To demonstrate the histone-defined enhancer classifier can predict VISTA enhancers, we removed the regions of VISTA limb enhancers that overlap Cotney et al. 2013 limb H3K27ac regions from the VISTA set and the regions of limb H3K27ac regions that overlap VISTA from the H3K27ac set to ensure no overlapping regions between training and testing. There are 96 human VISTA limb enhancers left and 32 mouse VISTA limb enhancers. Because of the small number of mouse enhancers, we only applied the human limb H3K27ac classifier to predict the human VISTA limb enhancers.

### Spectrum kernel SVM classification

An SVM is a discriminative classifier that learns a hyperplane to separate the positive and negative training data in feature space. We used the *k*-mer spectrum kernel to quantify sequence features for the SVM [78]. Training, classification, evaluation, and the computation of features weights were performed with the kebabs R package (v1.4.1) [49]. We used the default kernel normalization to the unit sphere, considered reverse complements separately, used the cosine similarity. We initially performed a grid search with *k* = 5 and C in the range of 1, 15, 50, 100, 1000 using the human liver enhancer dataset. We found that the performance of the SVMs in cross-validation is robust in C = 1,15 with cross validation errors of 0.2610, 0.2608 and ROC AUCs of 0.8213, 0.8209. We chose C = 15 for training SVMs in human and other species. The good performance in cross-validation runs suggest the SVMs are well regularized with C = 15 and any slight over-estimation of performance would result in an underestimation of cross-species generalization. Due to the imbalanced training dataset, we set class weights of 10 for the positives and 1 for the negatives to increase the penalty on misclassification of positives. We report all analyses with *k* = 5, but classifier performance and generalization were similar for *k* = 4–7 (0.81, 0.82, 0.82, 0.82, respectively for liver).

To evaluate classifier performance within-species, we performed ten-fold cross validation. In other words, for each set of positives and negatives, the entire data set was randomly partitioned into ten independent sets that maintained the ratio of positives and negatives. Positives and negatives from nine of the ten sets were then used to train the classifier, the trained classifier was then applied to the remaining partition, and these predictions were used to evaluate the classifier. This process was performed ten times, testing each partition once. To summarize performance, we averaged the auROC and auPR over the ten runs. For cross-species classification, we trained on the whole dataset in the training species and evaluated the performance on the test species.

We also evaluated more flexible models, such as the mismatch [49,78] and gappy pair kernels [49,79], These *k*-mer-based prediction models are similar to the spectrum kernel, but the mismatch kernel allows a maximum mismatch of *m* nucleotides in the *k*-mer and the gappy pair kernel considers pairs of *k*-mers with maximum gap of length *m* between them. For comparison, we trained the gappy pair kernel with *k* = 2, *m* = 1 and mismatch kernel with *k* = 5, *m* = 1 to compare with the 5-mer spectrum kernel. The mismatch and gappy pair kernels did not significantly increase the performance (auROCs of 0.82 and 0.82, respectively for liver) and are less interpretable than the *k*-mer spectra (S2 Fig). It is possible that other parameter settings could yield slightly improved performance, but the resulting models would be more difficult to interpret, and optimizing performance was not the goal of our study.

### Transcription factor motif analysis

5-mers were matched to known TF binding motifs in the JASPAR 2014 Core vertebrate database [57] using the TOMTOM package with default parameters [80]. The sharing of 5-mers and TFs across species was visualized using UpSetR [81].

### Transcription factor expression data

For the human TF expression analysis, we obtained RNA-seq data for TFs across 12 tissues from the Gene Expression Atlas (https://expressionatlas.org/hg19/adult/). Genes with non-zero FPKM (Fragments Per Kilobase of transcript per Million mapped reads) in a tissue were considered as expressed. For all the other species, we obtained the expression of TFs from Berthelot et al. 2017 [82]. The mouse TF expression in Berthelot et al. 2017 was first reported in Rudolph et al. 2016[83], so we obtained the mouse gene expression from in Rudolph et al. 2016.

### Convolutional neural network (CNN) classifier training and interpretation

Because of the fixed-length input of CNNs and the challenges of training CNNs using unbalanced datasets, we used the center 3000 bp (approximately the median length) of liver enhancers in six selected species as the positive training sequences and the same number of length matched random genomic regions in the corresponding species as negative training sequences During data preparation, we partitioned the data into training (80%), validation (10%), and hold-out testing sets (10%).

A typical convolutional neural network consists of convolutional layers, max-pooling layers, fully connected layers, and an output layer. To determine the CNN structure (S25 Fig), we defined a hyperparameter space, including a range of learning rates (0.0001, 0.0005, 0.001), number of convolutional layers (3 to 5), number of neurons in each layer (32, 64, 128, 256, 512) of the window size of the filters (4, 8, 16), the window size of pooling (0, 4), and the regularization strength (dropout fraction 0–1). We trained 100 CNN models on human liver enhancers with the training dataset and selected the structure of CNN based on the smallest loss on the validation set using keras 2.0.8 [84] with hyperparameters suggested by the Tree-structured Parzen Estimator (TPE) approach implemented in the hyperopt [85] library. Then, we trained the enhancer CNN model with the best human CNN structure in the other five species, but different regularization strengths, 30 times in order to find the best performing CNN model for each species based on the loss of validation set. The performance of within-species prediction is reported based on the auROC of predicting the hold-out testing set of the training species and the performance of cross-species prediction is reported based on the auROC of predicting all data in the testing species. To prevent the model overfitting the training data, we used an early stopping strategy during the training, together with dropout layers, and data partitioning. More specifically, we monitored the loss on the validation set and stopped the training process if the validation loss ceased decreasing.

To interpret the first layer of the human liver CNN, we forward propagated sequences in the human liver validation dataset through the CNN and selected the sequence patches that maximally activate each neuron (> 0.5 maximum activation value of the neuron) in the first layer. Then, we converted the resulting sets of sequence patches to position weight matrices (PWMs) and mapped the PWMs to human TF motifs from the HOCOMOCO v11 [86] database using TOMTOM with default parameters [80].

### Comparison of CNNs to *k*-mer SVM, polynomial kernel SVM, and gkm-SVM models

For comparison to the performance of CNNs, we trained gkm-SVM [87], polynomial kernel SVM and a 5-mer spectrum kernel SVM on the same balanced dataset as the CNNs. For gkm-SVM, we split the training data into 90% training set and 10% testing set. Then we trained gkm-SVMs with default parameters (wgkm kernel, l = 11, k = 7, d = 3) for 2 different Cs (0.1, 1). With C of 0.1, the training of gkm-SVM took 15.5 hours on a machine with a 2.4 GHz Intel Xeon CPU E5-2630 v3, 8 cores, and 2 CPUs; with C of 1, the training took 2 days and 13.5 hours. We report the performance of gkm-SVM on prediction of the testing set. For the polynomial kernel SVM, we split the training data into 90% training set and 10% testing set for each species. Then, we trained 5-mer 2^nd^ degree polynomial kernel SVMs on the *k*-mer spectrum of the training sequences in human. We selected C of 0.001 and performed the training of the polynomial kernel SVMs for every species. We report the performance of the polynomial kernel SVMs on the prediction of testing set. For the 5-mer spectrum SVMs, the performance of within-species prediction is reported based on the average auROC of ten-fold cross validation and the performance of cross-species prediction is reported based on the auROC of predicting all data in the testing species. The better performance of CNNs compared to the SVMs is not driven by differences in the testing set. When using the exact same training and testing set of the human liver enhancer dataset, the 5-mer SVM achieved ROC AUC of 0.782 and PR AUC of 0.756, which are very similar to the average performance over cross-validation folds: ROC AUC of 0.783, PR AUC of 0.761. Similarly, the gkm-SVM achieved ROC AUC of 0.767 and PR AUC of 0.749, which are similar to the reported performance of gkm-SVM in cross validation: ROC AUC of 0.763 and PR AUC of 0.745.

## Supporting information

S1 Fig Precision-recall (PR) curves for the classification of enhancers vs. the genomic background (non-GC-controlled). (a) Classification of liver enhancers in six diverse mammals: human (Hsap, experiment 1), macaque (Mmul, experiment 8), mouse (Mmus, experiment 15), cow (Btau, experiment 22), dog (Cfam, experiment 29), and opossum (Mdom, experiment 36). (b) Classification of developing limb enhancers in human (experiment 147), macaque (experiment 151), and mouse (experiment 155). (c) Classification of developing brain enhancers in human (experiment 165), macaque (experiment 169), and mouse (experiment 173). (d) Generalization of the human-trained liver enhancer classifier to the other five mammals (experiment 1s-6). The cross-validation PR curve for a classifier trained and tested on human is included for reference. (e) Generalization of the human-trained limb enhancer classifier to macaque and mouse (experiment 147–149). (f) Generalization of the human-trained brain enhancer classifier to macaque and mouse (experiment 165–167). AUC values are given after the species name. The cross-validation PR curve for a classifier trained and tested on human is included for reference.
(PDF)

S2 Fig Performance for the classification of human liver enhancers vs. the genomic background with 5-mer spectrum, gappy pair (experiment 145, k = 2, m = 1) and mismatch kernel (experiment 146, k = 5, m = 1). (a) Receiver operating characteristics (ROC) curves. (b) Precision-recall (PR) curves. AUC values are given after the method name.
(PDF)

S3 Fig The predictions of enhancer classifiers (not-GC-controlled) trained in different species were strongly correlated. Scatter plots showing the correlation between scores assigned to human enhancers by the human-trained classifier and the classifiers trained on other species: (a) Human (Hsap, experiment 1) vs. Macaque (Mmul, experiment 7). (b) Human vs. Mouse (Mmus, experiment 13) (c) Human vs. Cow (Btau, experiment 19) (d) Human vs. Dog (Cfam, experiment 25) (e) Human vs. Opossum (Mdom, experiment 31). Each dot represents a human liver enhancer sequence. The enhancer score assigned by the human-trained classifier is plotted on the x-axis, and the score assigned by the classifier trained on the other specified species is plotted on the y-axis. The color indicates the GC content. Correlation is quantified by Spearman’s rank correlation coefficient (ρ). (f) The GC content distribution of liver enhancers in human, macaque, mouse, cow, dog, and opossum. Human, macaque, cow and dog enhancers have a similar GC distribution. Mouse and opossum have less variation in GC content and are depleted of high GC enhancers compared to the other species. (g) The GC content of human enhancers is positively correlated with the scores assigned by the human-trained classifier (Pearson’s r = 0.54, P<2.2e-16).
(PDF)

S4 Fig Evaluation of between species liver enhancer classification (non-GC-controlled, experiment 1–36). (a) auROC. (b) auPR. (c) Raw decrease of cross-species auROC compared to within species auROC. (d) Raw decrease of cross-species auPR compared to within species auPR.
(PDF)

S5 Fig Neutral sequence divergence is inversely correlated with the cross-species prediction accuracy. (a) Correlation of relative auROCs from the non-GC-controlled classifiers (experiments 1–36) with sequence divergence. Spearman’s rho is –0.4 (*P* = 0.14). (b) Correlation of relative auPRs from the non-GC-controlled classifiers (experiments 1–36) with sequence divergence. Spearman’s rho is –0.38 (*P* = 0.16). Both correlations increased significantly when accounting for GC-content in the classifiers (S15 Fig). Sequence divergence is quantified as the expected number of substitutions per neutrally evolving site as derived from four-fold degenerate sites in codons in the UCSC Genome Browser’s100-way multiple species alignments (Methods). To determine the relative auROC/PR for each pair of species, the mean was taken across the two classifiers when applied cross-species (i.e., the relative auROC/PR from the human classifier applied to mouse and the relative auROC/PR mouse classifier applied to human were averaged).
(PDF)

S6 Fig Evaluation of between species limb enhancer classification (non-GC-controlled, experiment 147–155). (a) auROC. (b) auPR. (c) Raw decrease of cross-species auROC compared to within species auROC. (d) Raw decrease of cross-species auPR compared to within species auPR.
(PDF)

S7 Fig Evaluation of between species brain enhancer classification (non-GC-controlled, experiment 165–173). (a) auROC. (b) auPR. (c) Raw decrease of cross-species auROC compared to within species auROC. (d) Raw decrease of cross-species auPR compared to within species auPR.
(PDF)

S8 Fig Evaluation of between human (Hsap) and mouse (Mmus) VISTA enhancer classification tasks (non-GC-controlled, experiment 189–232). The number of enhancers in each tissue is indicated in brackets. (a) Forebrain enhancers (Human, 312; Mouse, 85) (b) Midbrain (Human, 259; Mouse 69) (c) Hindbrain (Human, 239; Mouse 58) (d) Heart (Human, 97; Mouse, 120) (e) Branchial arch (Human, 73; Mouse, 73). (f) Limb (Human 168; Mouse, 84). The human classifier usually generalized better than mouse classifiers. This may be due to the larger sample size of human enhancers in most of the tissues.
(PDF)

S9 Fig Evaluation of between species liver enhancer classification (GC-controlled, experiment 37–72). (a) auROC (b) auPR (c) Raw decrease of cross-species auROC compared to within species auROC (d) Raw decrease of cross-species auPR compared to within species auPR.
(PDF)

S10 Fig Evaluation of between species limb enhancer classification (GC-controlled, experiment 156–164). (a) auROC (b) auPR (c) Raw decrease of cross-species auROC compared to within species auROC (d) Raw decrease of cross-species auPR compared to within species auPR. (e) Relative cross-species auROC (f) Relative cross-species auPR.
(PDF)

S11 Fig Evaluation of between species brain enhancer classification (GC-controlled, experiment 174–182). (a) auROC (b) auPR (c) Raw decrease of cross-species auROC compared to within species auROC (d) Raw decrease of cross-species auPR compared to within species auPR. (e) Relative cross-species auROC (f) Relative cross-species auPR.
(PDF)

S12 Fig Evaluation of between human (Hsap) and mouse (Mmus) VISTA enhancer GC-controlled classification tasks (experiments 189–212). The species on the x-axis are the training species, and the species on the y-axis are the testing species. (a) Forebrain enhancers (Human, 312; Mouse, 85) (b) Midbrain (Human, 259; Mouse 69) (c) Hindbrain (Human, 239; Mouse 58) (d) Heart (Human, 97; Mouse, 120) (e) Branchial arch (Human, 73; Mouse, 73). (f) Limb (Human 168; Mouse, 84). The human classifier usually generalized better than mouse classifiers. This may be due to the larger sample size of human enhancers in most of the tissues.
(PDF)

S13 Fig The predictions of enhancer classifiers trained in different species are strongly correlated (GC-controlled analysis). Scatter plots showing the correlation between scores assigned to human enhancers by the human-trained classifier and the classifiers trained on other species in GC-controlled analysis: (a) Human (experiment 37) vs. Macaque (experiment 43). (b) Human vs. Mouse (experiment 49) (c) Human vs. Cow (experiment 55) (d) Human vs. Dog (experiment 61) (e) Human vs. Opossum (experiment 67). Each dot represents a human liver enhancer sequence. The enhancer score assigned by the human-trained classifier is plotted on the x-axis, and the score assigned by the classifier trained on the other specified species is plotted on the y-axis. The color indicates the GC content. The correlation between enhancer scores produced by different species classifiers is quantified by Spearman’s rank correlation coefficient (ρ). (f) The GC content of human enhancers has low correlation with the scores assigned by the human-trained classifier (Pearson’s r = –0.076, P<2.2e-16).
(PDF)

S14 Fig Evaluation of between species liver enhancer classification using flanking regions as negatives (experiments 372–407). We evaluated the ability of the 5-mer spectrum classifier to distinguish enhancers from flanking regions and the ability of these classifiers to generalize across species: (a) auROC, (b) auPR, (c) relative auROC, (d) relative auPR. We defined the flanking region of an enhancer as 10 times its length on either side. We then randomly selected 10 negative regions of same length as the enhancer that did not overlap other enhancers from the candidate flanking regions. Classifiers were then applied across species. The classifiers performed similarly to the GC-controlled classifiers and generalized very well across species. The dog classifier had much lower performance and generalization than the other classifiers. This could indicate differences in the sequence similarity of regulatory neighborhoods in dogs or be due to the quality of the dog genome assembly.
(PDF)

S15 Fig Neutral sequence divergence is significantly inversely correlated with the GC-controlled cross-species prediction accuracy. (a) Correlation of relative auROCs from the GC-controlled classifiers (experiments 37–72) with sequence divergence. Spearman’s rho is –0.72 (*P* = 0). (b) Correlation of relative auPRs from the GC-controlled classifiers (experiments 37–72) with sequence divergence. Spearman’s rho is –0.52 (*P* = 0.05). Sequence divergence is quantified as the number of substitutions per neutrally evolving site as derived from four-fold degenerate sites in codons in the UCSC Genome Browser’s100-way multiple species alignments (Methods). To determine the relative auROC/PR for each pair of species, the mean was taken across the two classifiers when applied cross-species (i.e., the relative auROC/PR from the human classifier applied to mouse and the relative auROC/PR mouse classifier applied to human were averaged).
(PDF)

S16 Fig Classifiers trained on enhancers lacking repetitive elements generalize across species (experiments 73–108). In liver enhancers from each species, we identified those that did not overlap a repetitive element (Methods). The vast majority of enhancers overlapped at least one repetitive element, leaving at total of 966 (human), 1321 (macaque), 914 (mouse), 2772 (cow), 451 (dog), 556 (opossum) enhancers. Classifiers trained on these ‘repeat-free’ enhancers generalized well across species as measured by (a) raw auROC and (b) relative auROC. Surprisingly, classifiers trained in other species better predicted dog and opossum enhancers than the dog and opossum trained classifiers. This is likely a consequence of the small training sets remaining for dog and opossum; these two species had the fewest liver enhancers without repeat overlap (451 and 556, respectively, while the other species each had at least 900 remaining).
(PDF)

S17 Fig Evaluation of between-species liver enhancer classification, controlling for both GC-content and proportion of overlap with repetitive elements (experiments 109–144). The random genomic background was matched for both GC-content and the proportion overlap with repetitive elements. Classifiers were then applied cross species. Classifiers were predictive of enhancers in other species by both (a) auROC (b) auPR. The opossum classifier generalized less well across species than the classifiers trained on other species, likely due to its low genome assembly quality.
(PDF)

S18 Fig Enhancer classifiers generalize more accurately across the same tissue in different species than across different tissues in the same species (relative auPRs). (a) The human-trained liver classifier obtains better performance when applied to liver enhancers from other species (gray dots) than when applied to enhancers from other human tissues. This also holds for GC-controlled analyses, with the exception of predicting enhancers active in the gastric mucosa. (b) In the not-GC-controlled analysis, the cross-species performance is significantly better than the cross-tissue (roadmap) performance (*P* = 0.00015, Mann Whitney U test) and the cross-tissue (Villar, Cotney, Reilly) performance (*P* = 4.9E-05). This also holds true for the GC-controlled analysis. The cross-species performance is significantly better than the cross-tissue (roadmap) performance (*P* = 0.049) and the cross-tissue (Villar, Cotney, Reilly) performance (*P* = 7.58E-08).
(PDF)

S19 Fig TFs matched by the top positive k-mers between the human classifier and other species are more similar than those between the human liver tissue and other Roadmap tissues (GC-controlled negatives). For each pair of SVM classifiers, the Jaccard similarity of the top positive k-mer-mapped TFs is plotted.
(PDF)

S20 Fig The DNA sequence patterns most predictive of liver enhancer activity across species matched a common set of transcription factors (non-GC-controlled). Of the TFs matched by the top 5-mers from each non-GC-controlled liver classifier (experiments 1, 8, 15, 22, 29, 36), 27 are shared by all six species.
(PDF)

S21 Fig CNNs perform substantially better than 5-mer spectrum SVMs, 5-mer polynomial kernel SVMs, and 11-mer gkm-SVMs across C values. We evaluate the performance of SVMs across a range of C values (0.001 to 15, x-axis, experiments 348–354, 366–371) and compare it with the CNN model (experiment 275, hyper-parameter selection is described in the Methods). (a) Comparison of auROCs between different classifiers. (b) Comparison of auPRs between different classifiers.
(PDF)

S22 Fig Evaluation of between species liver enhancer classification with balanced, non-GC-controlled negative set using convolutional neural networks (CNNs) and SVMs. (a) Raw auROCs of cross-species enhancer predictions using CNNs, experiments 275–310. (b) Relative auROC of cross-species enhancer predictions using CNNs, experiments 275–310. (c) Raw auPRs of cross-species enhancer predictions using CNNs, experiments 275–310. (d) Relative auPRs of cross-species enhancer predictions using CNNs, experiments 275–310. (e) Raw auROCs of cross-species enhancer predictions using SVMs, experiments 311–346. (f) Relative auROCs of cross-species enhancer predictions using SVMs, experiments 311–346. (g) Raw auPRs of cross-species enhancer predictions using SVMs, experiments 311–346. (h) Relative auPRs of cross-species enhancer predictions using SVMs, experiments 311–346.
(PDF)

S23 Fig The CNNs trained on GC-controlled, repeat-controlled enhancer datasets with orthologous enhancers removed performed better than the 5-mer spectrum SVMs trained on the same data and generalized worse across species (experiments 354–365). (a) The auROCs of CNN models were substantially better than the 5-mer SVM models in each species. The error bars give the standard error of ten-fold cross-validation for the SVM models. We removed the enhancer orthologs between each pair of human and another species. For instance, “Hsap without Mmus” means human enhancers with mouse enhancer orthologs removed from consideration. (b) The auPRs of CNN models were substantially better than the 5-mer SVM models in each species. The error bars give the standard error of ten-fold cross-validation for the SVM models. (c) The relative auROCs of the CNN models applied across species are consistently lower than for the 5-mer spectrum SVMs applied across the same species. (d) The relative auPRs of the CNN models applied across species are consistently lower than for the 5-mer spectrum SVMs applied across the same species. This suggests that the CNN models did not generalize as well across species as the SVM models.
(PDF)

S24 Fig The 5-mer polynomial kernel SVMs trained on enhancers and random genomic regions (experiments 408–443) performed similarly to 5-mer spectrum SVMs within and across species. (a) The auROCs of 5-mer polynomial kernel SVMs are similar to 5-mer spectrum SVMs within species and are substantially worse than the CNNs in each species. The error bars give the standard error of ten-fold cross-validation for the 5-mer spectrum SVM models. (b) The auPRs of 5-mer polynomial kernel SVMs are similar to 5-mer spectrum SVMs within species and are substantially worse than the CNNs in each species. (c) The relative auROCs of the 5-mer polynomial kernel SVMs applied across species are similar to the 5-mer SVMs applied across the same species. (d) The relative auPRs of the 5-mer polynomial kernel SVMs applied across species are similar to the 5-mer SVMs applied across the same species. This suggests that the 5-mer polynomial kernel SVMs generalized as well across species as the simpler SVM models.
(PDF)

S25 Fig The convolutional neural network (CNN) structure for training CNN classifiers of liver enhancers. (PDF)

S1 Table Summary of performance of all classification tasks. (XLS)

S2 Table Liver expression of the shared TF motifs in the liver GC-controlled analysis. (XLSX)

S3 Table The sharing of the TF motifs matched by the top 5% positive 5-mers from each classifier in liver, limb and brain. (XLSX)
