# Supplementary figures and images for: Prediction of gene regulatory enhancers across species reveals evolutionarily conserved sequence properties
Source: PLoS Comput Biol. 2018 Oct 4;14(10):e1006484. doi: 10.1371/journal.pcbi.1006484 (PMC6191148; doi:10.1371/journal.pcbi.1006484)

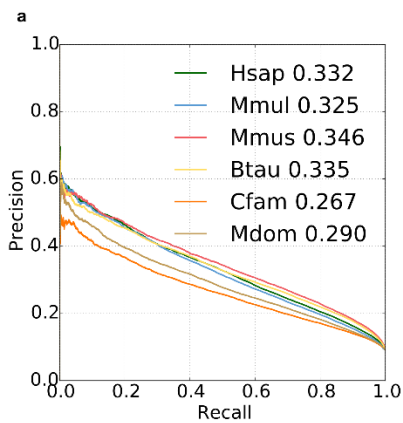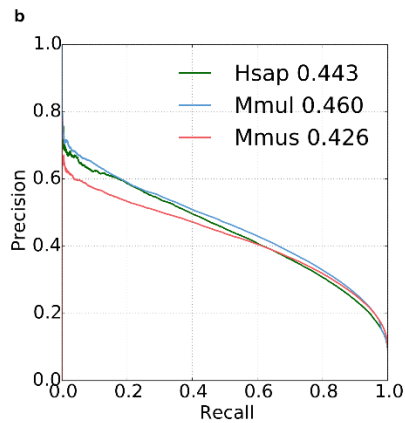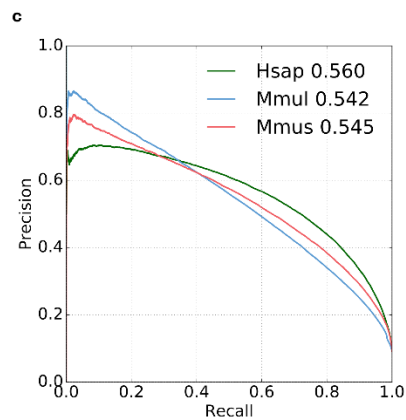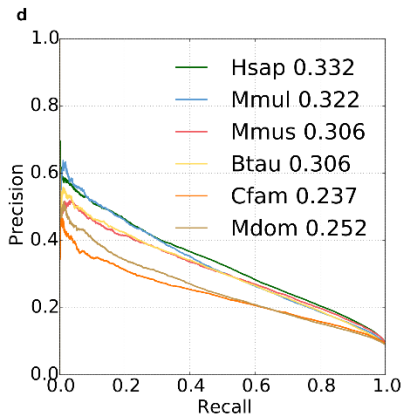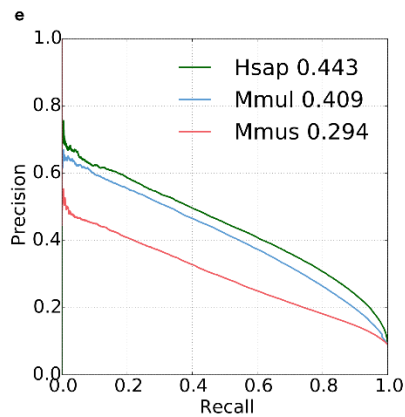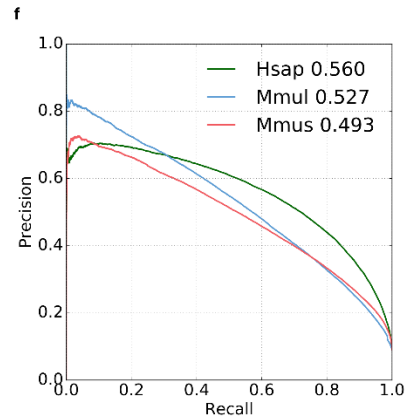

Supplement: S1 Fig — (a) Classification of liver enhancers in six diverse mammals: human (Hsap, experiment 1), macaque (Mmul, experiment 8), mouse (Mmus, experiment 15), cow (Btau, experiment 22), dog (Cfam, experiment 29), and opossum (Mdom, experiment 36). (b) Classification of developing limb enhancers in human (experiment 147), macaque (experiment 151), and mouse (experiment 155). (c) Classification of developing brain enhancers in human (experiment 165), macaque (experiment 169), and mouse (experiment 173). (d) Generalization of the human-trained liver enhancer classifier to the other five mammals (experiment 1s-6). The cross-validation PR curve for a classifier trained and tested on human is included for reference. (e) Generalization of the human-trained limb enhancer classifier to macaque and mouse (experiment 147–149). (f) Generalization of the human-trained brain enhancer classifier to macaque and mouse (experiment 165–167). AUC values are given after the species name. The cross-validation PR curve for a classifier trained and tested on human is included for reference. (PDF) [file pcbi.1006484.s001.pdf]

**a**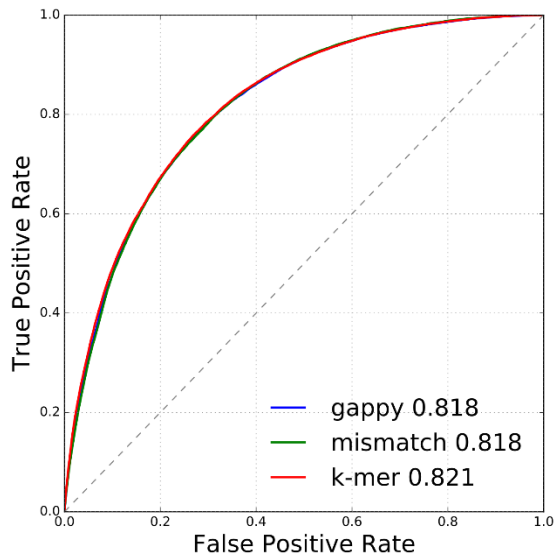**b**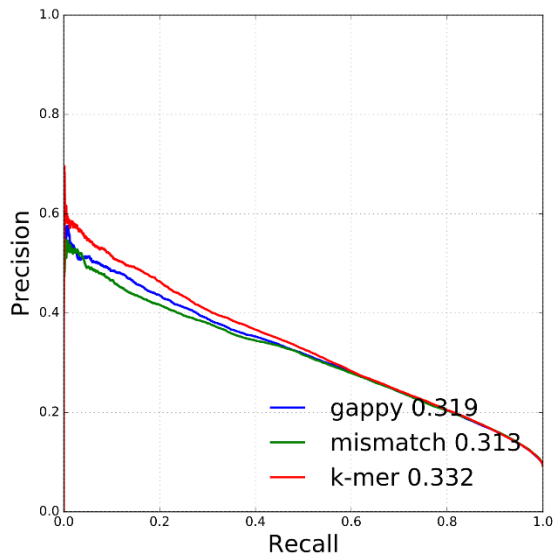

Supplement: S2 Fig — (a) Receiver operating characteristics (ROC) curves. (b) Precision-recall (PR) curves. AUC values are given after the method name. (PDF) [file pcbi.1006484.s002.pdf]

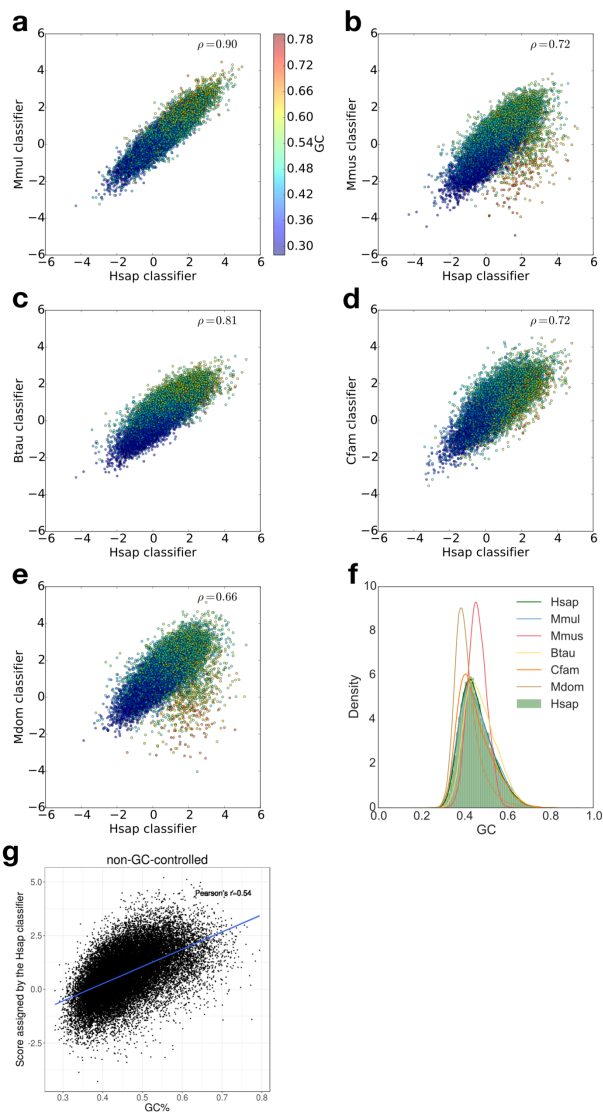

Supplement: S3 Fig — Scatter plots showing the correlation between scores assigned to human enhancers by the human-trained classifier and the classifiers trained on other species: (a) Human (Hsap, experiment 1) vs. Macaque (Mmul, experiment 7). (b) Human vs. Mouse (Mmus, experiment 13) (c) Human vs. Cow (Btau, experiment 19) (d) Human vs. Dog (Cfam, experiment 25) (e) Human vs. Opossum (Mdom, experiment 31). Each dot represents a human liver enhancer sequence. The enhancer score assigned by the human-trained classifier is plotted on the x-axis, and the score assigned by the classifier trained on the other specified species is plotted on the y-axis. The color indicates the GC content. Correlation is quantified by Spearman’s rank correlation coefficient (ρ). (f) The GC content distribution of liver enhancers in human, macaque, mouse, cow, dog, and opossum. Human, macaque, cow and dog enhancers have a similar GC distribution. Mouse and opossum have less variation in GC content and are depleted of high GC enhancers compared to the other species. (g) The GC content of human enhancers is positively correlated with the scores assigned by the human-trained classifier (Pearson’s r = 0.54, P<2.2e-16). (PDF) [file pcbi.1006484.s003.pdf]

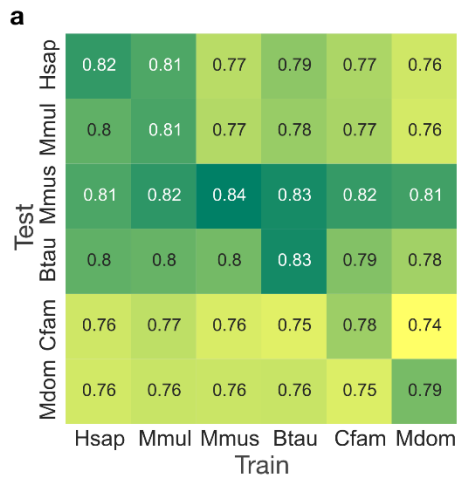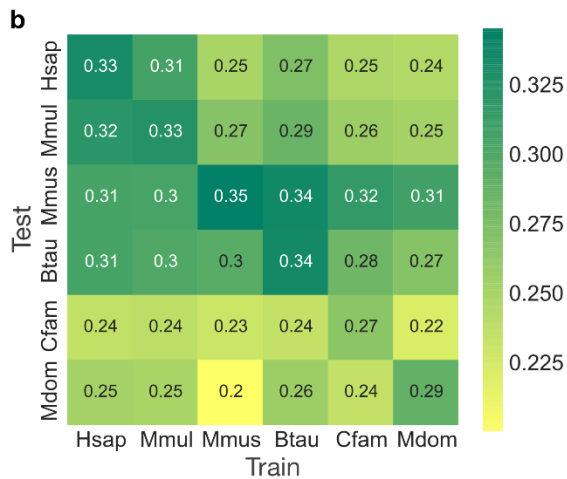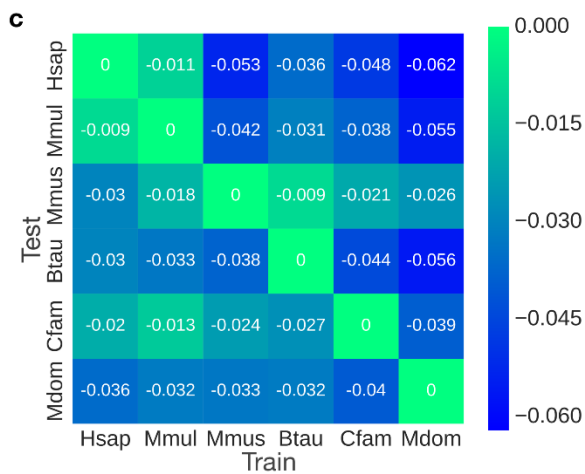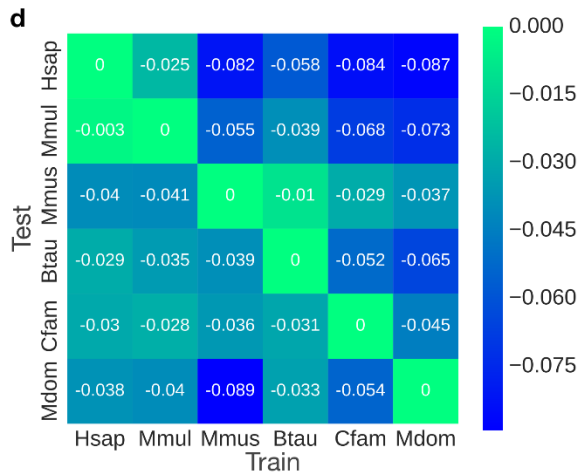

Supplement: S4 Fig — (a) auROC. (b) auPR. (c) Raw decrease of cross-species auROC compared to within species auROC. (d) Raw decrease of cross-species auPR compared to within species auPR. (PDF) [file pcbi.1006484.s004.pdf]

**a** Not-GC-controlled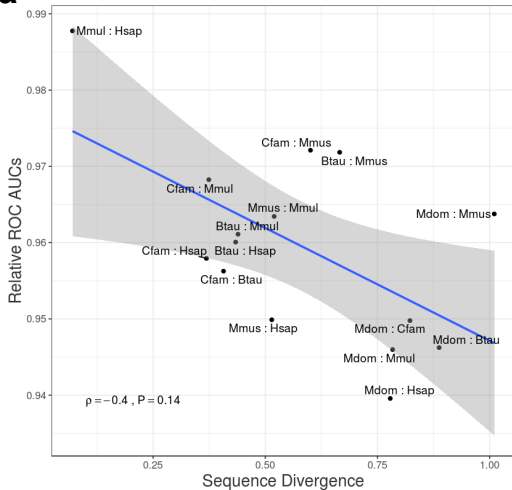**b** Not-GC-controlled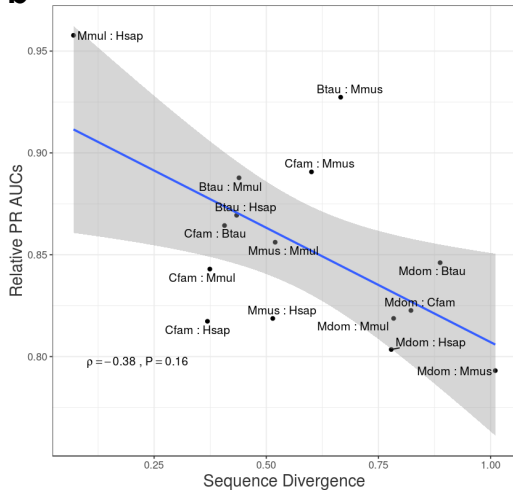

Supplement: S5 Fig — (a) Correlation of relative auROCs from the non-GC-controlled classifiers (experiments 1–36) with sequence divergence. Spearman’s rho is –0.4 (P = 0.14). (b) Correlation of relative auPRs from the non-GC-controlled classifiers (experiments 1–36) with sequence divergence. Spearman’s rho is –0.38 (P = 0.16). Both correlations increased significantly when accounting for GC-content in the classifiers (S15 Fig). Sequence divergence is quantified as the expected number of substitutions per neutrally evolving site as derived from four-fold degenerate sites in codons in the UCSC Genome Browser’s100-way multiple species alignments (Methods). To determine the relative auROC/PR for each pair of species, the mean was taken across the two classifiers when applied cross-species (i.e., the relative auROC/PR from the human classifier applied to mouse and the relative auROC/PR mouse classifier applied to human were averaged). (PDF) [file pcbi.1006484.s005.pdf]

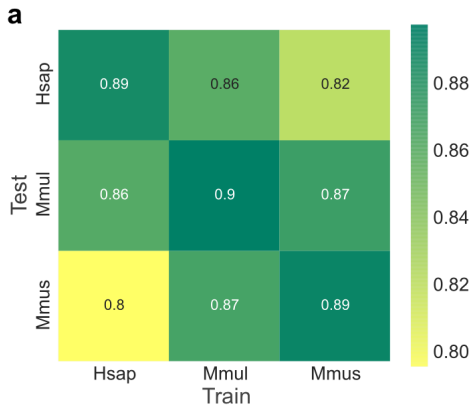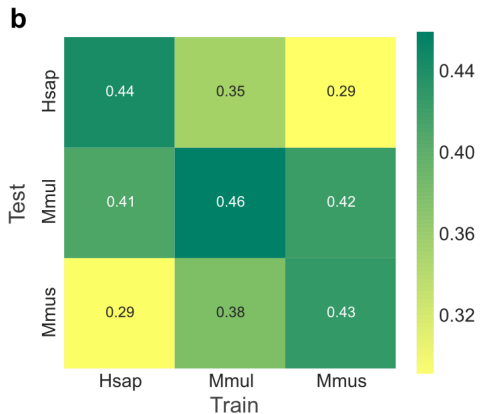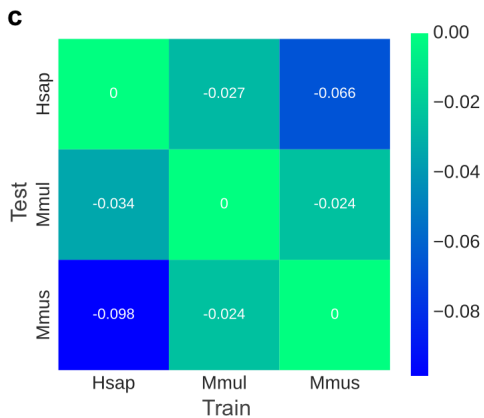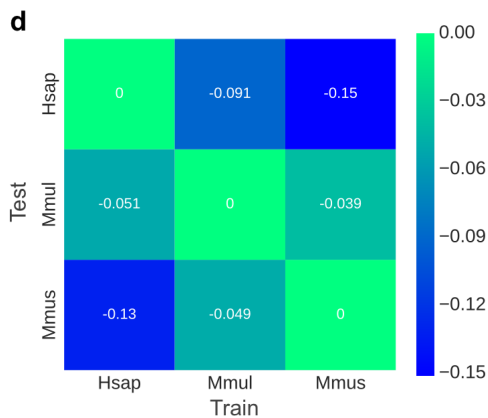

Supplement: S6 Fig — (a) auROC. (b) auPR. (c) Raw decrease of cross-species auROC compared to within species auROC. (d) Raw decrease of cross-species auPR compared to within species auPR. (PDF) [file pcbi.1006484.s006.pdf]

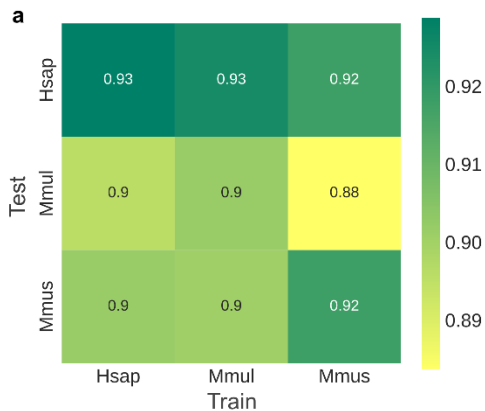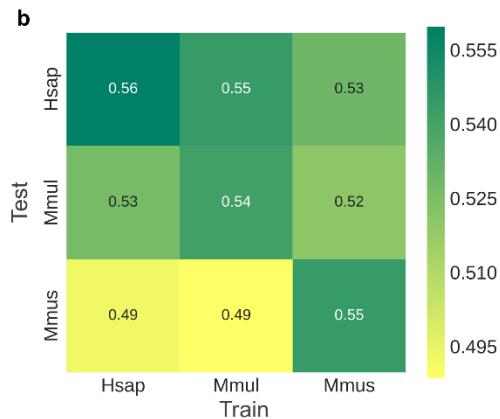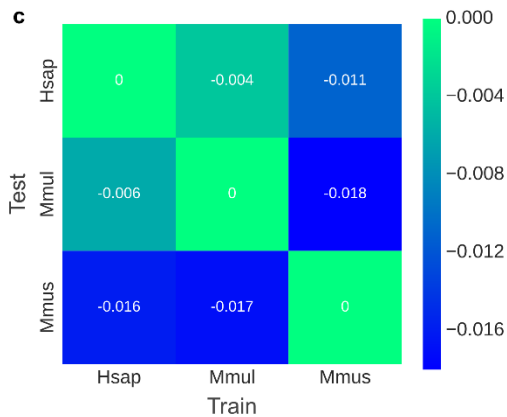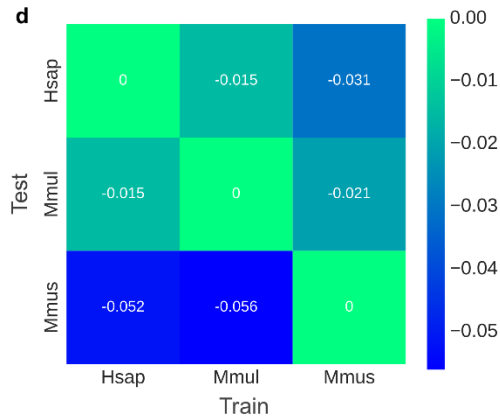

Supplement: S7 Fig — (a) auROC. (b) auPR. (c) Raw decrease of cross-species auROC compared to within species auROC. (d) Raw decrease of cross-species auPR compared to within species auPR. (PDF) [file pcbi.1006484.s007.pdf]

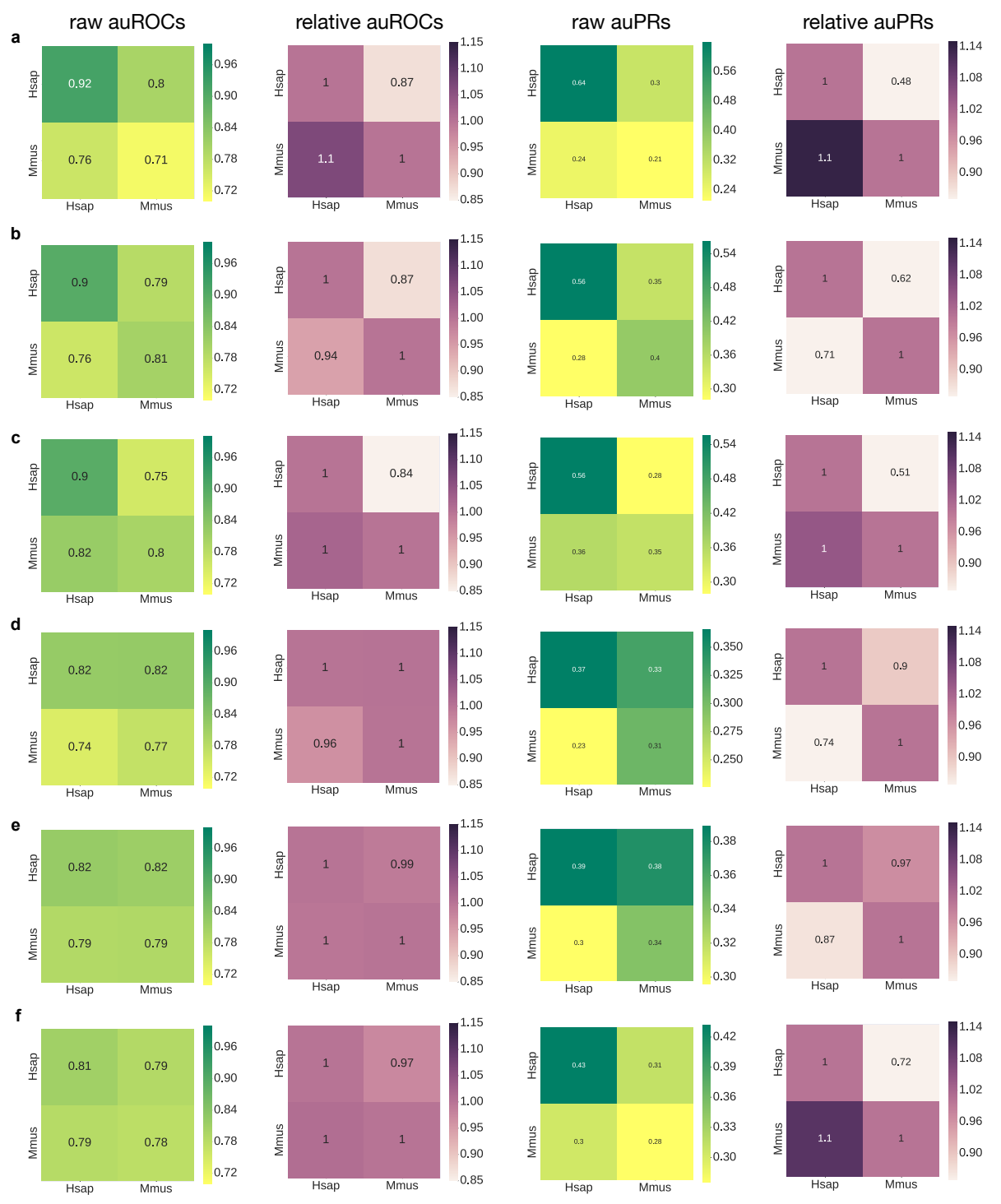

Supplement: S8 Fig — The number of enhancers in each tissue is indicated in brackets. (a) Forebrain enhancers (Human, 312; Mouse, 85) (b) Midbrain (Human, 259; Mouse 69) (c) Hindbrain (Human, 239; Mouse 58) (d) Heart (Human, 97; Mouse, 120) (e) Branchial arch (Human, 73; Mouse, 73). (f) Limb (Human 168; Mouse, 84). The human classifier usually generalized better than mouse classifiers. This may be due to the larger sample size of human enhancers in most of the tissues. (PDF) [file pcbi.1006484.s008.pdf]

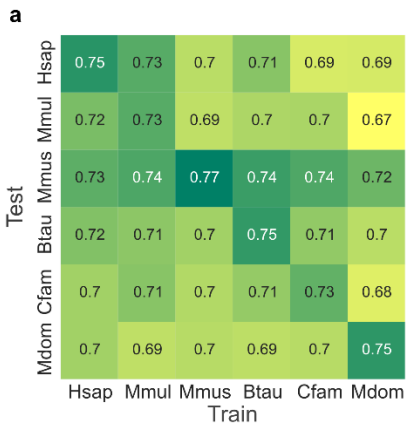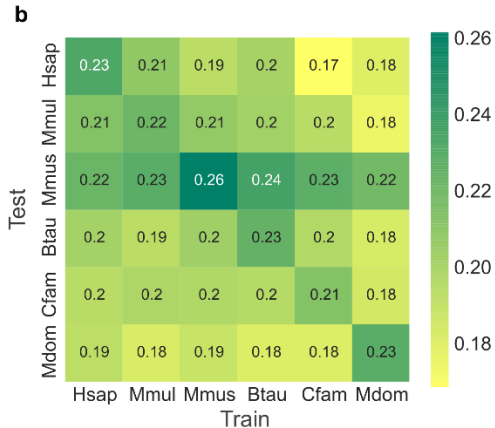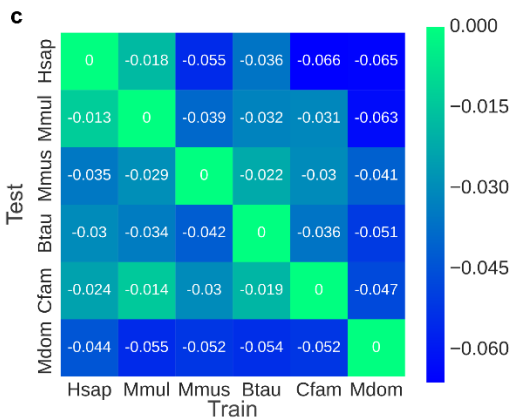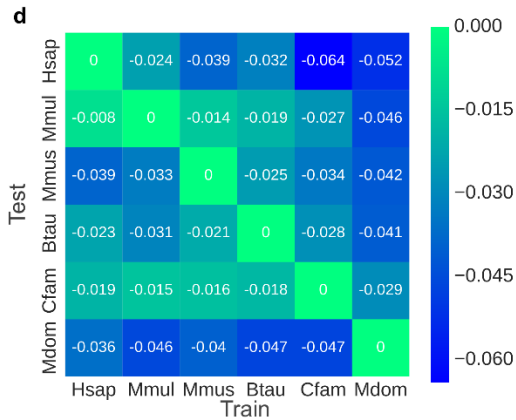

Supplement: S9 Fig — (a) auROC (b) auPR (c) Raw decrease of cross-species auROC compared to within species auROC (d) Raw decrease of cross-species auPR compared to within species auPR. (PDF) [file pcbi.1006484.s009.pdf]

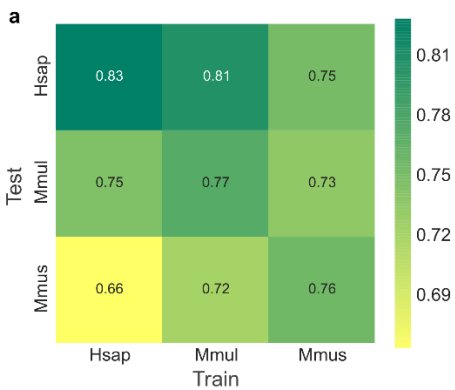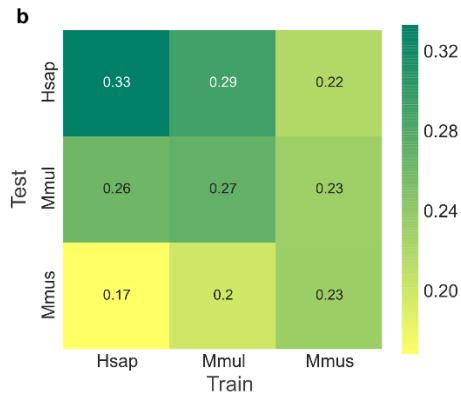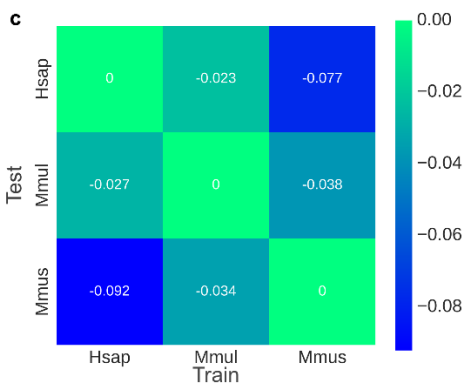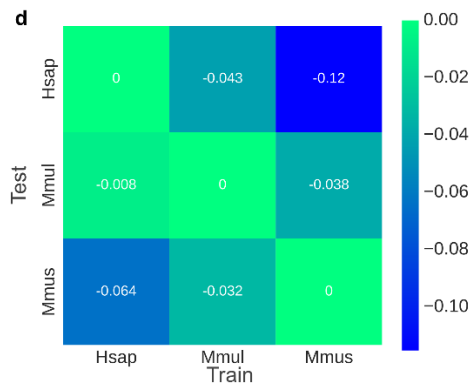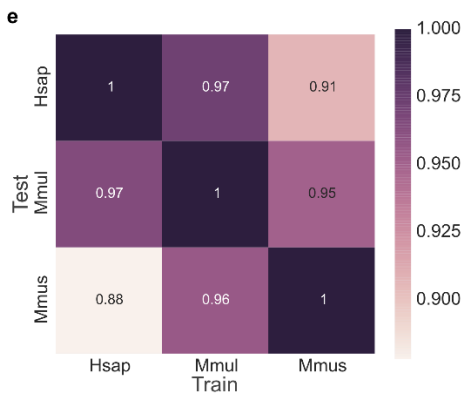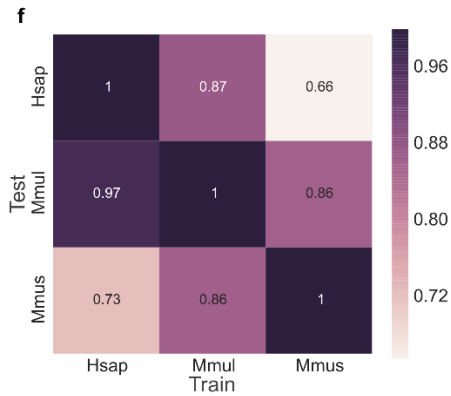

Supplement: S10 Fig — (a) auROC (b) auPR (c) Raw decrease of cross-species auROC compared to within species auROC (d) Raw decrease of cross-species auPR compared to within species auPR. (e) Relative cross-species auROC (f) Relative cross-species auPR. (PDF) [file pcbi.1006484.s010.pdf]

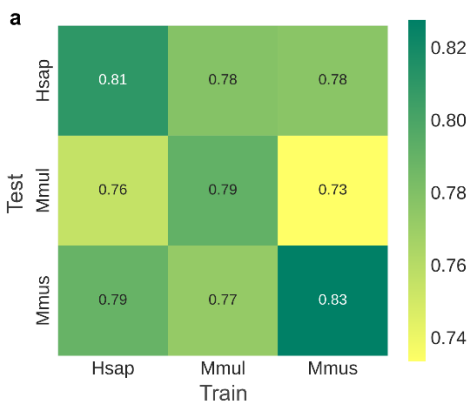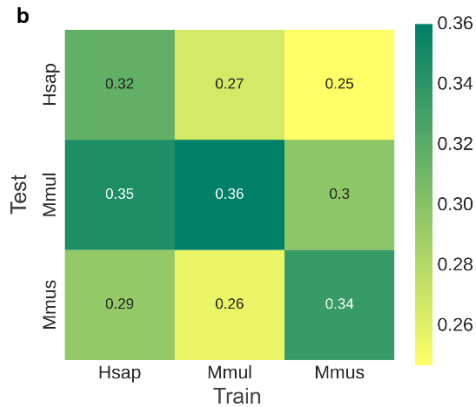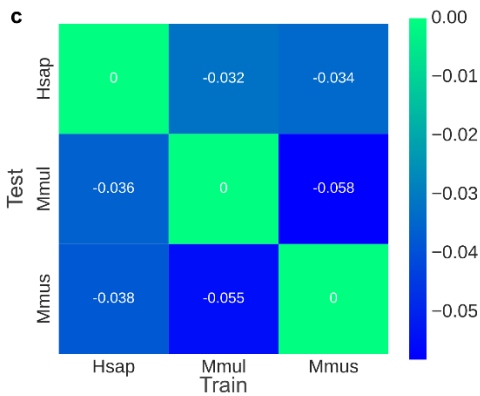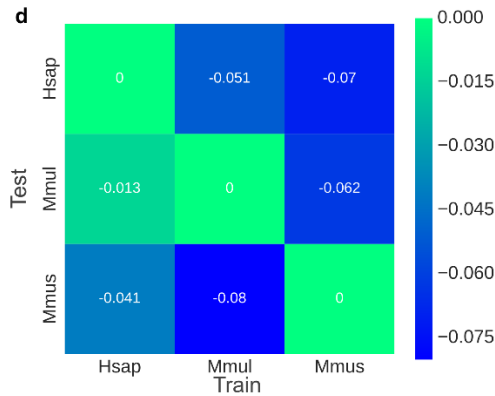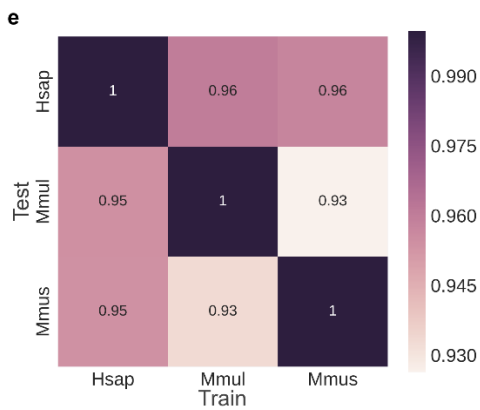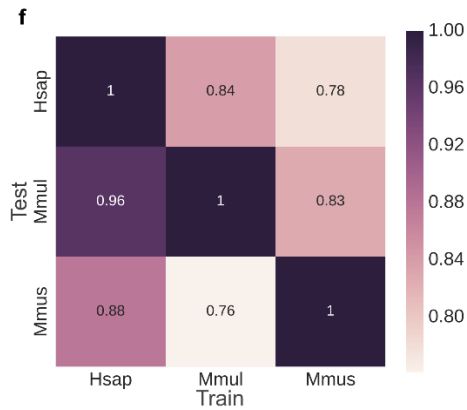

Supplement: S11 Fig — (a) auROC (b) auPR (c) Raw decrease of cross-species auROC compared to within species auROC (d) Raw decrease of cross-species auPR compared to within species auPR. (e) Relative cross-species auROC (f) Relative cross-species auPR. (PDF) [file pcbi.1006484.s011.pdf]

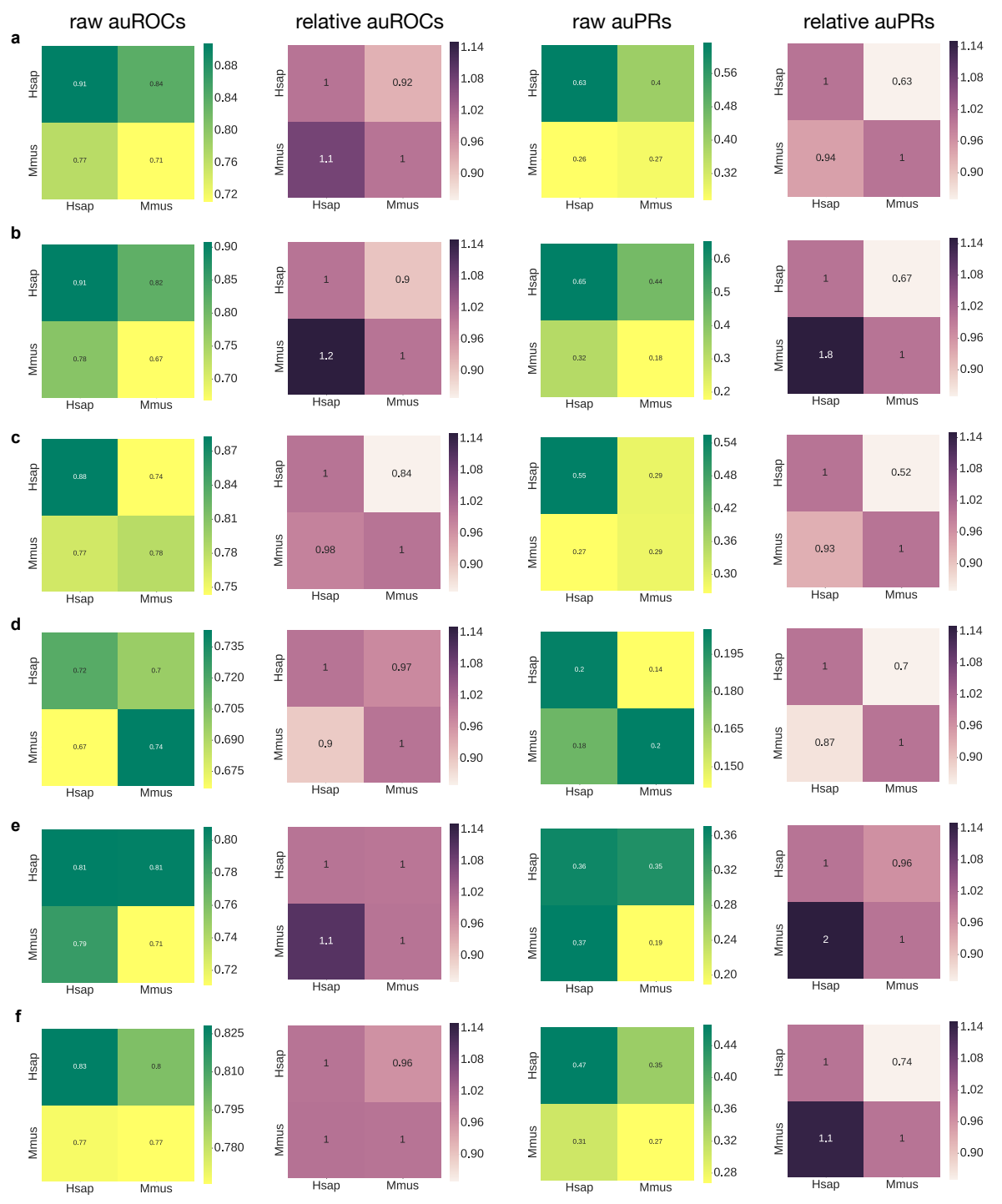

Supplement: S12 Fig — The species on the x-axis are the training species, and the species on the y-axis are the testing species. (a) Forebrain enhancers (Human, 312; Mouse, 85) (b) Midbrain (Human, 259; Mouse 69) (c) Hindbrain (Human, 239; Mouse 58) (d) Heart (Human, 97; Mouse, 120) (e) Branchial arch (Human, 73; Mouse, 73). (f) Limb (Human 168; Mouse, 84). The human classifier usually generalized better than mouse classifiers. This may be due to the larger sample size of human enhancers in most of the tissues. (PDF) [file pcbi.1006484.s012.pdf]

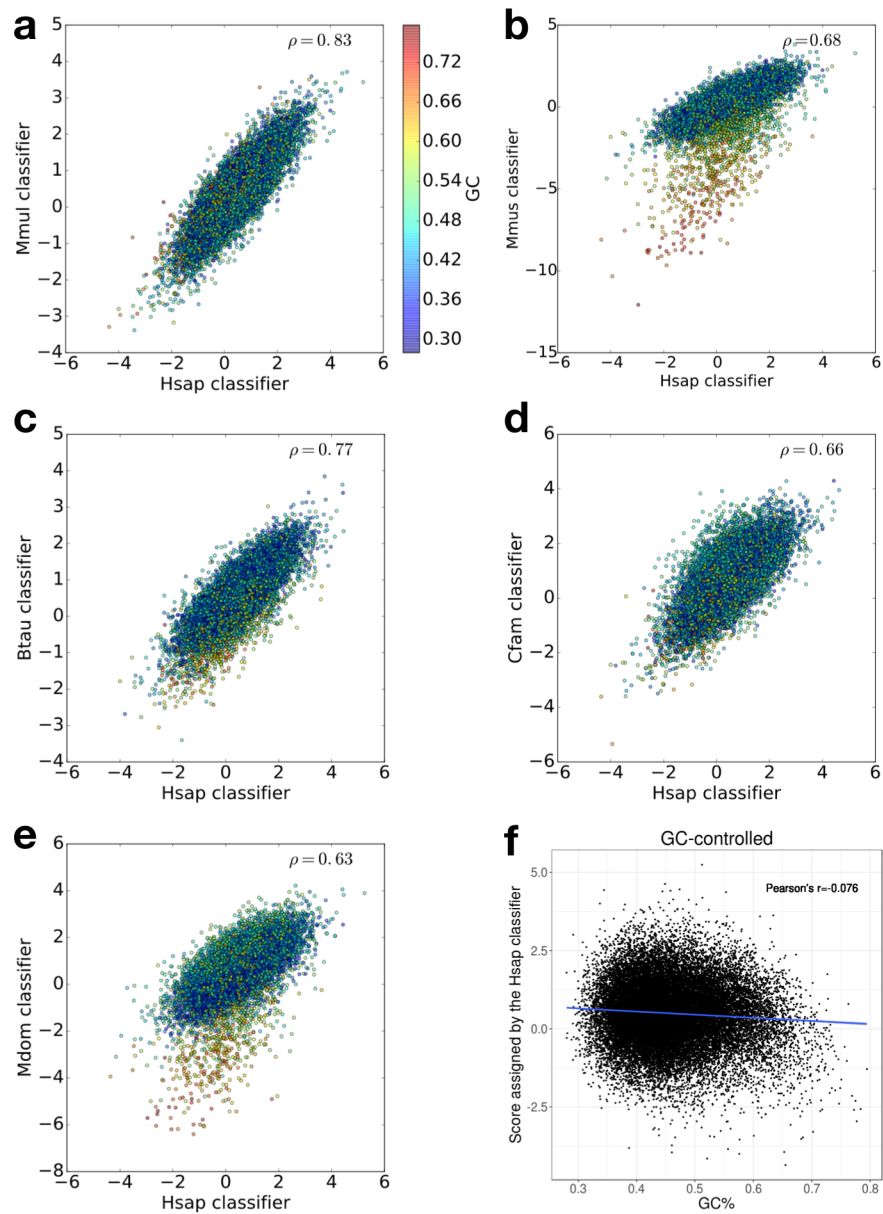

Supplement: S13 Fig — Scatter plots showing the correlation between scores assigned to human enhancers by the human-trained classifier and the classifiers trained on other species in GC-controlled analysis: (a) Human (experiment 37) vs. Macaque (experiment 43). (b) Human vs. Mouse (experiment 49) (c) Human vs. Cow (experiment 55) (d) Human vs. Dog (experiment 61) (e) Human vs. Opossum (experiment 67). Each dot represents a human liver enhancer sequence. The enhancer score assigned by the human-trained classifier is plotted on the x-axis, and the score assigned by the classifier trained on the other specified species is plotted on the y-axis. The color indicates the GC content. The correlation between enhancer scores produced by different species classifiers is quantified by Spearman’s rank correlation coefficient (ρ). (f) The GC content of human enhancers has low correlation with the scores assigned by the human-trained classifier (Pearson’s r = –0.076, P<2.2e-16). (PDF) [file pcbi.1006484.s013.pdf]

**a**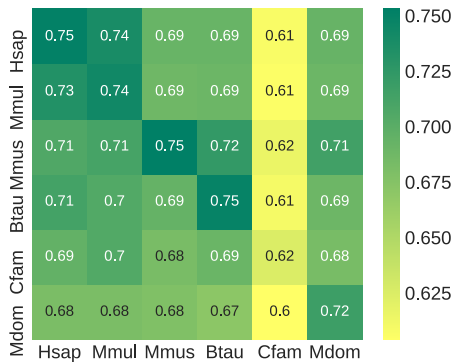**b**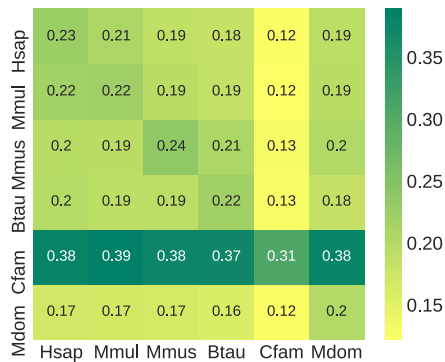**c**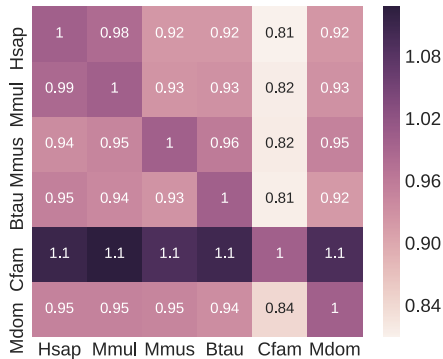**d**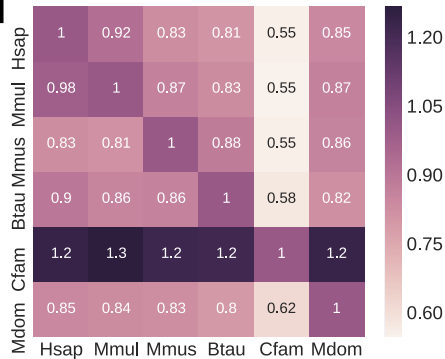

Supplement: S14 Fig — We evaluated the ability of the 5-mer spectrum classifier to distinguish enhancers from flanking regions and the ability of these classifiers to generalize across species: (a) auROC, (b) auPR, (c) relative auROC, (d) relative auPR. We defined the flanking region of an enhancer as 10 times its length on either side. We then randomly selected 10 negative regions of same length as the enhancer that did not overlap other enhancers from the candidate flanking regions. Classifiers were then applied across species. The classifiers performed similarly to the GC-controlled classifiers and generalized very well across species. The dog classifier had much lower performance and generalization than the other classifiers. This could indicate differences in the sequence similarity of regulatory neighborhoods in dogs or be due to the quality of the dog genome assembly. (PDF) [file pcbi.1006484.s014.pdf]

**a**

GC-controlled

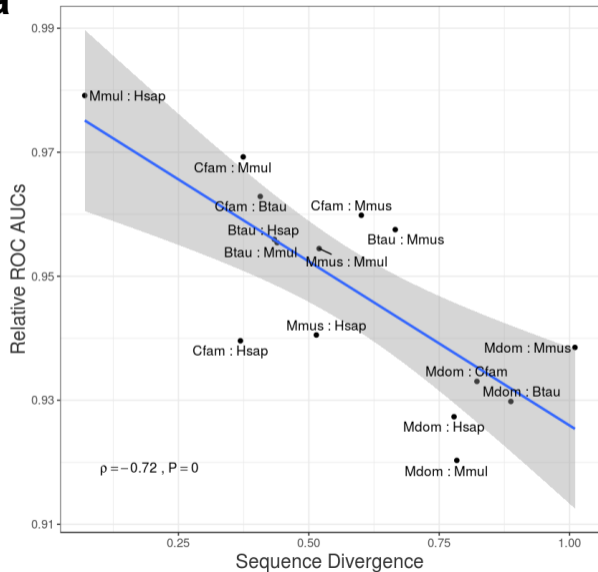**b**

GC-controlled

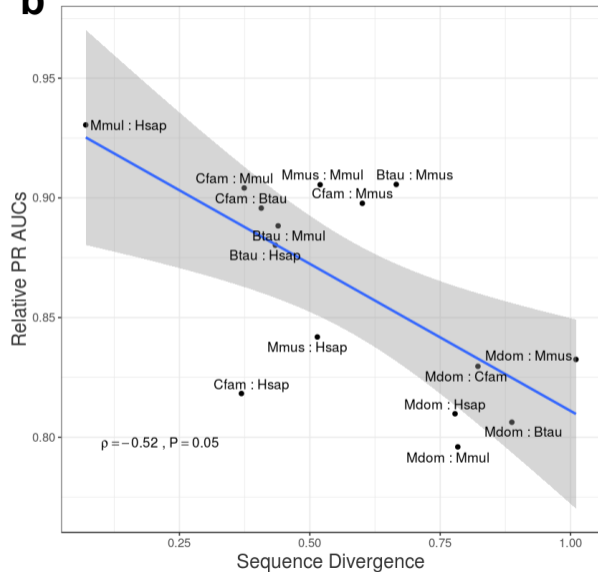

Supplement: S15 Fig — (a) Correlation of relative auROCs from the GC-controlled classifiers (experiments 37–72) with sequence divergence. Spearman’s rho is –0.72 (P = 0). (b) Correlation of relative auPRs from the GC-controlled classifiers (experiments 37–72) with sequence divergence. Spearman’s rho is –0.52 (P = 0.05). Sequence divergence is quantified as the number of substitutions per neutrally evolving site as derived from four-fold degenerate sites in codons in the UCSC Genome Browser’s100-way multiple species alignments (Methods). To determine the relative auROC/PR for each pair of species, the mean was taken across the two classifiers when applied cross-species (i.e., the relative auROC/PR from the human classifier applied to mouse and the relative auROC/PR mouse classifier applied to human were averaged). (PDF) [file pcbi.1006484.s015.pdf]

**a**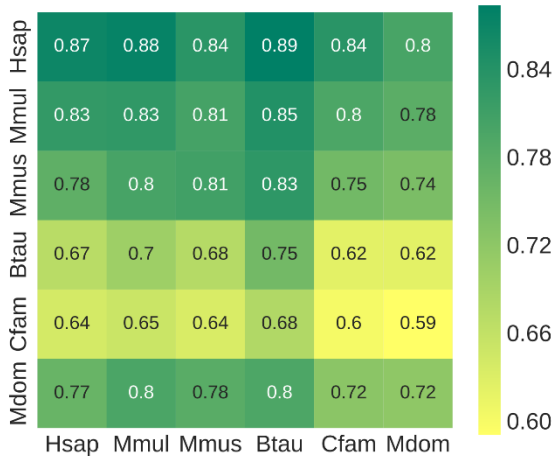**b**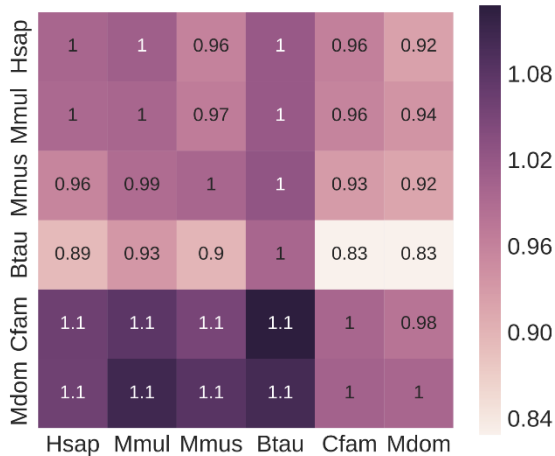

Supplement: S16 Fig — In liver enhancers from each species, we identified those that did not overlap a repetitive element (Methods). The vast majority of enhancers overlapped at least one repetitive element, leaving at total of 966 (human), 1321 (macaque), 914 (mouse), 2772 (cow), 451 (dog), 556 (opossum) enhancers. Classifiers trained on these ‘repeat-free’ enhancers generalized well across species as measured by (a) raw auROC and (b) relative auROC. Surprisingly, classifiers trained in other species better predicted dog and opossum enhancers than the dog and opossum trained classifiers. This is likely a consequence of the small training sets remaining for dog and opossum; these two species had the fewest liver enhancers without repeat overlap (451 and 556, respectively, while the other species each had at least 900 remaining). (PDF) [file pcbi.1006484.s016.pdf]

**a**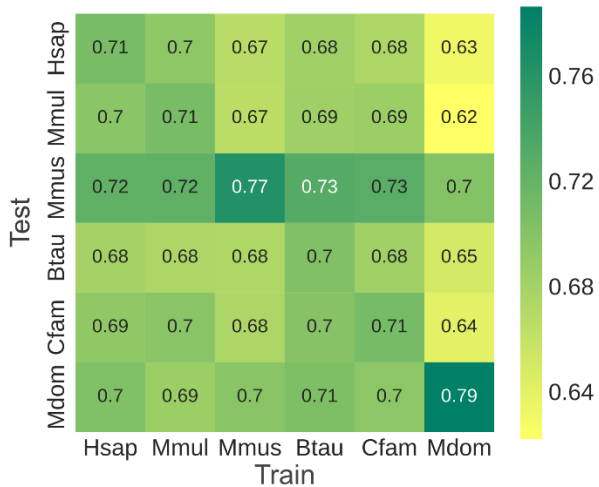**b**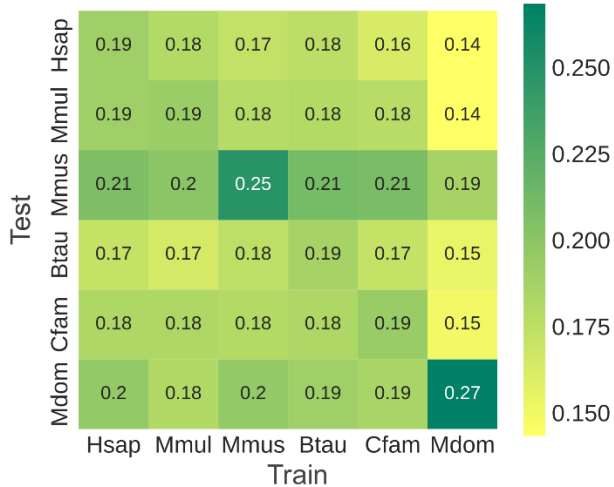

Supplement: S17 Fig — The random genomic background was matched for both GC-content and the proportion overlap with repetitive elements. Classifiers were then applied cross species. Classifiers were predictive of enhancers in other species by both (a) auROC (b) auPR. The opossum classifier generalized less well across species than the classifiers trained on other species, likely due to its low genome assembly quality. (PDF) [file pcbi.1006484.s017.pdf]

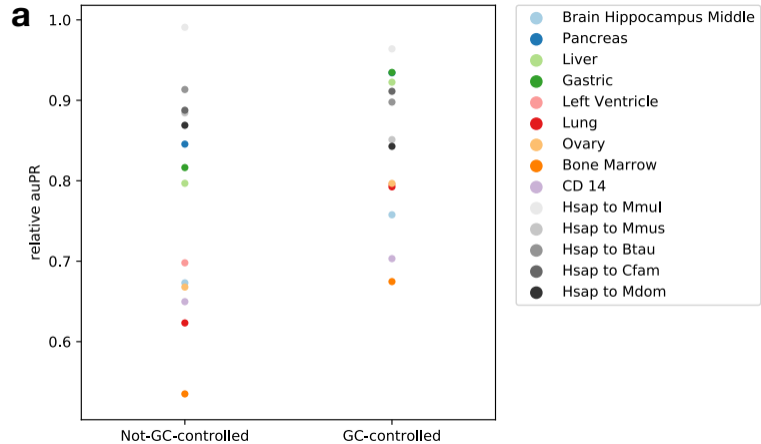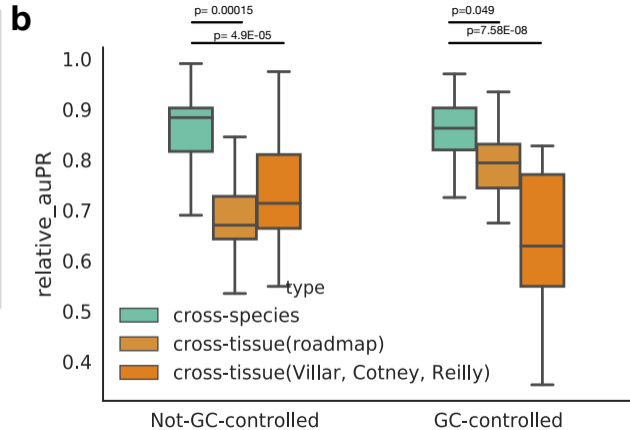

Supplement: S18 Fig — (a) The human-trained liver classifier obtains better performance when applied to liver enhancers from other species (gray dots) than when applied to enhancers from other human tissues. This also holds for GC-controlled analyses, with the exception of predicting enhancers active in the gastric mucosa. (b) In the not-GC-controlled analysis, the cross-species performance is significantly better than the cross-tissue (roadmap) performance (P = 0.00015, Mann Whitney U test) and the cross-tissue (Villar, Cotney, Reilly) performance (P = 4.9E-05). This also holds true for the GC-controlled analysis. The cross-species performance is significantly better than the cross-tissue (roadmap) performance (P = 0.049) and the cross-tissue (Villar, Cotney, Reilly) performance (P = 7.58E-08). (PDF) [file pcbi.1006484.s018.pdf]

# TF sharing

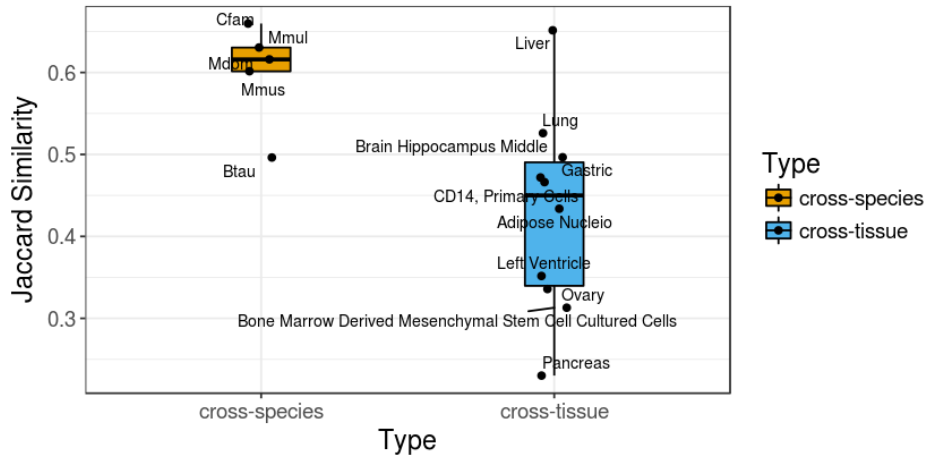

Supplement: S19 Fig — For each pair of SVM classifiers, the Jaccard similarity of the top positive k-mer-mapped TFs is plotted. (PDF) [file pcbi.1006484.s019.pdf]

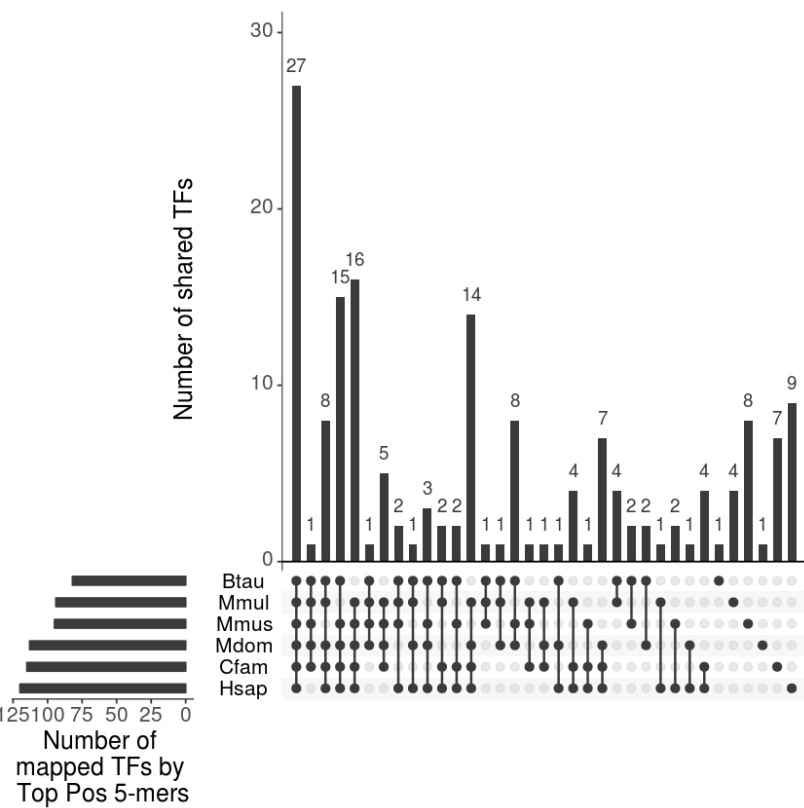

Supplement: S20 Fig — Of the TFs matched by the top 5-mers from each non-GC-controlled liver classifier (experiments 1, 8, 15, 22, 29, 36), 27 are shared by all six species. (PDF) [file pcbi.1006484.s020.pdf]

**a**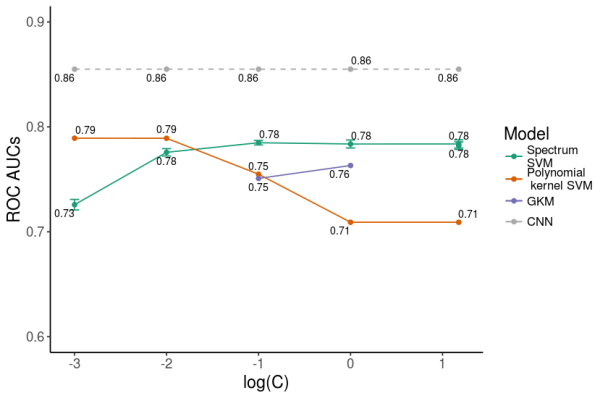**b**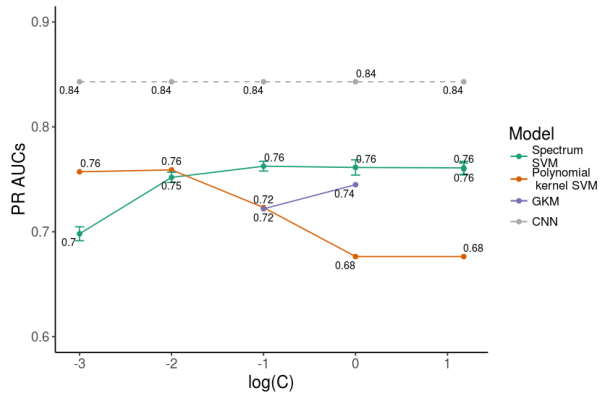

Supplement: S21 Fig — We evaluate the performance of SVMs across a range of C values (0.001 to 15, x-axis, experiments 348–354, 366–371) and compare it with the CNN model (experiment 275, hyper-parameter selection is described in the Methods). (a) Comparison of auROCs between different classifiers. (b) Comparison of auPRs between different classifiers. (PDF) [file pcbi.1006484.s021.pdf]

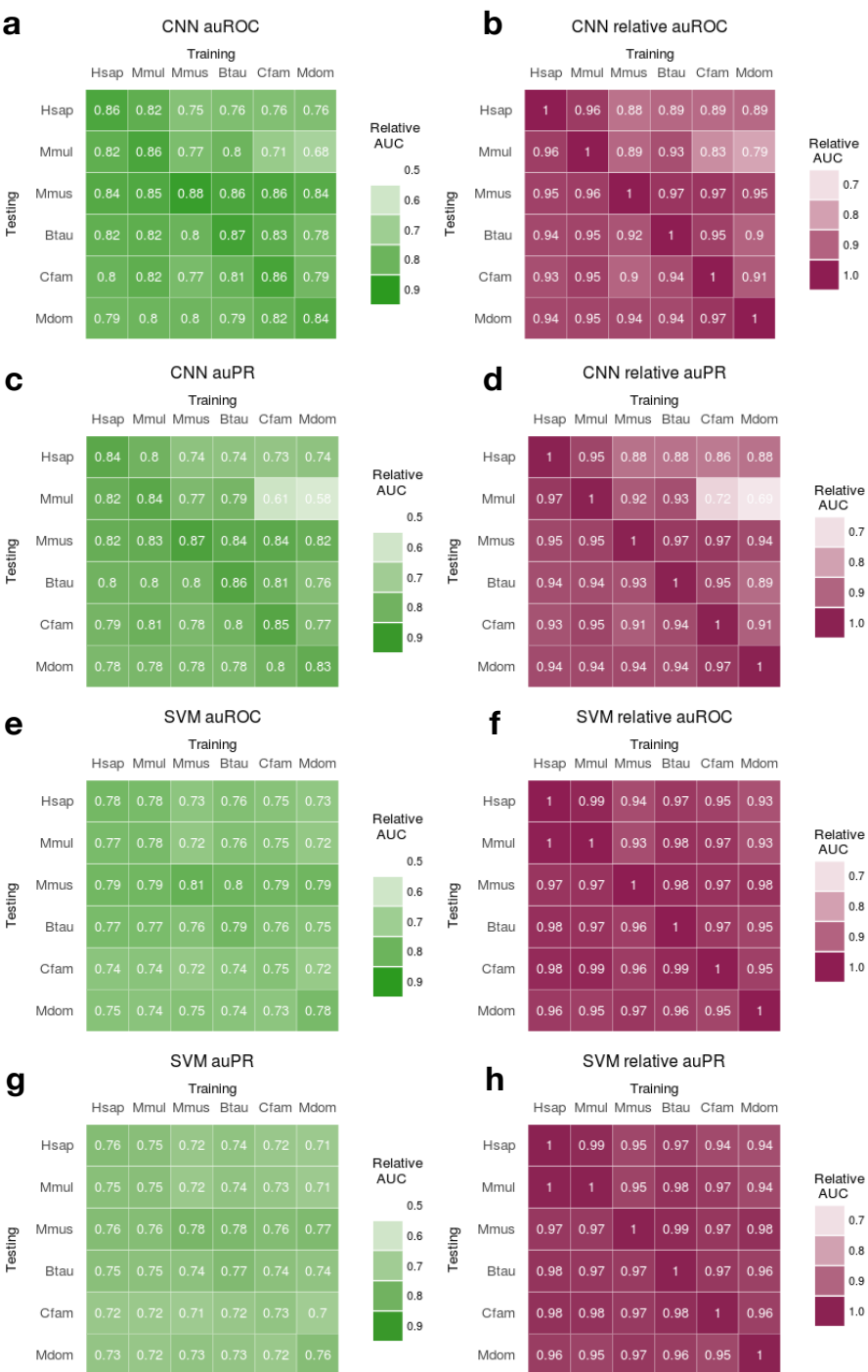

Supplement: S22 Fig — (a) Raw auROCs of cross-species enhancer predictions using CNNs, experiments 275–310. (b) Relative auROC of cross-species enhancer predictions using CNNs, experiments 275–310. (c) Raw auPRs of cross-species enhancer predictions using CNNs, experiments 275–310. (d) Relative auPRs of cross-species enhancer predictions using CNNs, experiments 275–310. (e) Raw auROCs of cross-species enhancer predictions using SVMs, experiments 311–346. (f) Relative auROCs of cross-species enhancer predictions using SVMs, experiments 311–346. (g) Raw auPRs of cross-species enhancer predictions using SVMs, experiments 311–346. (h) Relative auPRs of cross-species enhancer predictions using SVMs, experiments 311–346. (PDF) [file pcbi.1006484.s022.pdf]

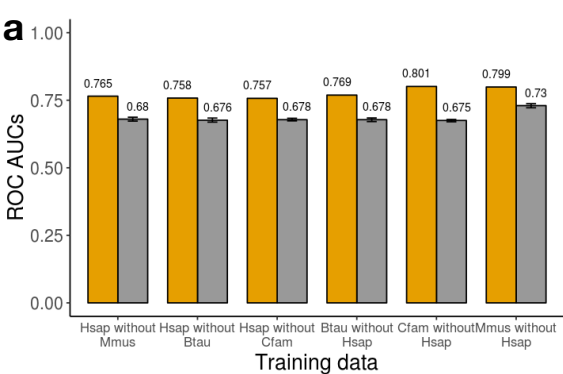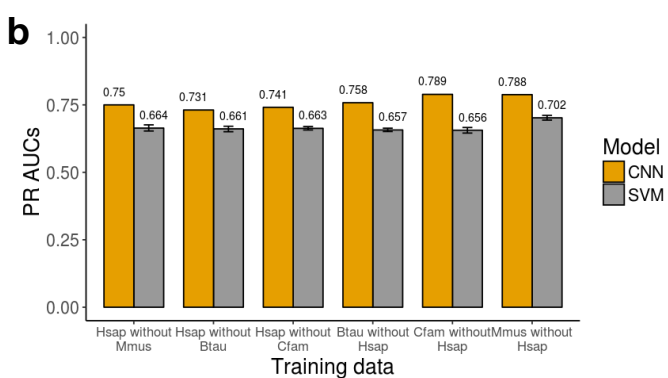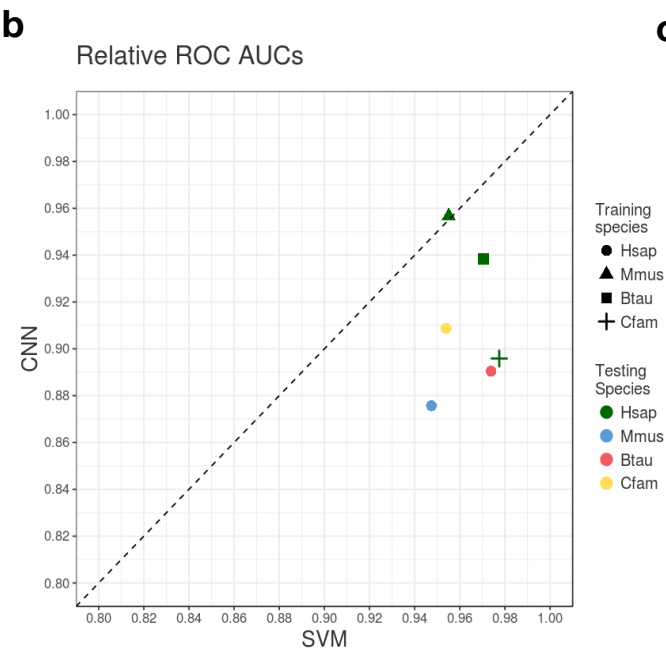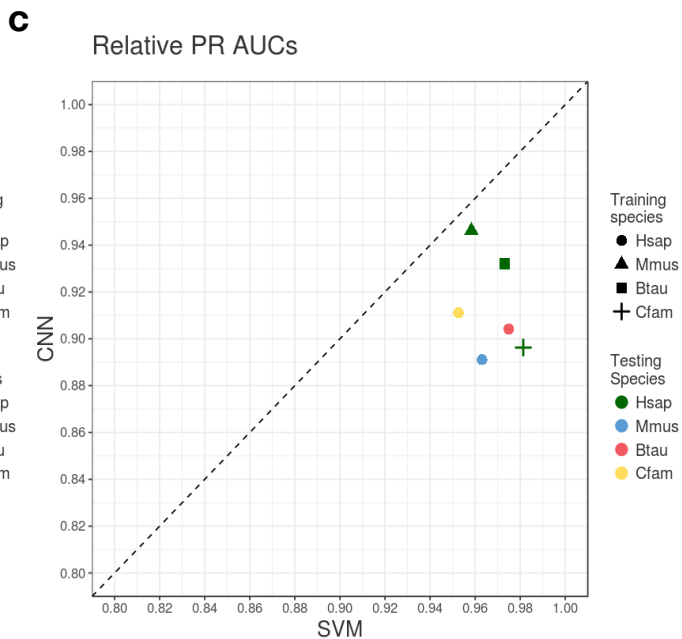

Supplement: S23 Fig — (a) The auROCs of CNN models were substantially better than the 5-mer SVM models in each species. The error bars give the standard error of ten-fold cross-validation for the SVM models. We removed the enhancer orthologs between each pair of human and another species. For instance, “Hsap without Mmus” means human enhancers with mouse enhancer orthologs removed from consideration. (b) The auPRs of CNN models were substantially better than the 5-mer SVM models in each species. The error bars give the standard error of ten-fold cross-validation for the SVM models. (c) The relative auROCs of the CNN models applied across species are consistently lower than for the 5-mer spectrum SVMs applied across the same species. (d) The relative auPRs of the CNN models applied across species are consistently lower than for the 5-mer spectrum SVMs applied across the same species. This suggests that the CNN models did not generalize as well across species as the SVM models. (PDF) [file pcbi.1006484.s023.pdf]

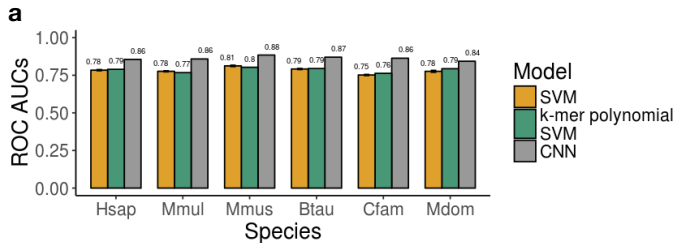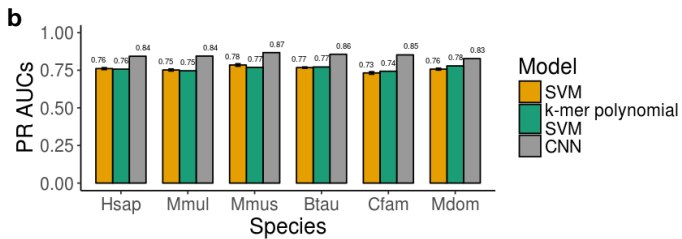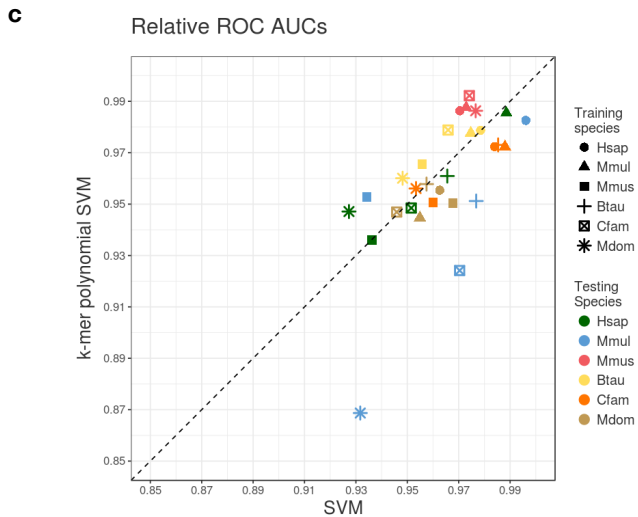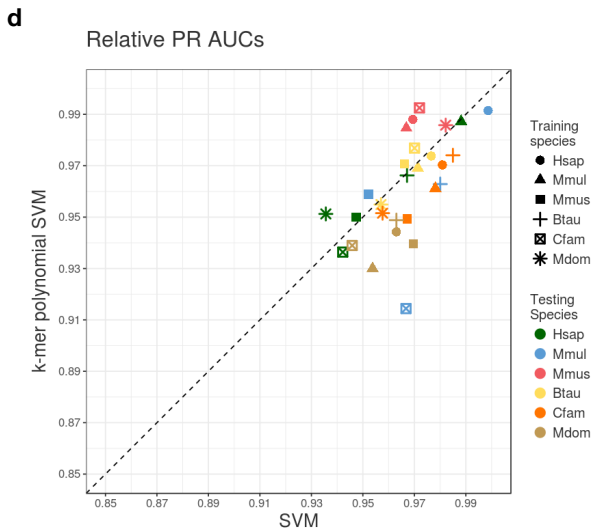

Supplement: S24 Fig — (a) The auROCs of 5-mer polynomial kernel SVMs are similar to 5-mer spectrum SVMs within species and are substantially worse than the CNNs in each species. The error bars give the standard error of ten-fold cross-validation for the 5-mer spectrum SVM models. (b) The auPRs of 5-mer polynomial kernel SVMs are similar to 5-mer spectrum SVMs within species and are substantially worse than the CNNs in each species. (c) The relative auROCs of the 5-mer polynomial kernel SVMs applied across species are similar to the 5-mer SVMs applied across the same species. (d) The relative auPRs of the 5-mer polynomial kernel SVMs applied across species are similar to the 5-mer SVMs applied across the same species. This suggests that the 5-mer polynomial kernel SVMs generalized as well across species as the simpler SVM models. (PDF) [file pcbi.1006484.s024.pdf]
