# Supplementary material for: Prediction of gene regulatory enhancers across species reveals evolutionarily conserved sequence properties
Source: PLoS Comput Biol. 2018 Oct 4;14(10):e1006484. doi: 10.1371/journal.pcbi.1006484 (PMC6191148; doi:10.1371/journal.pcbi.1006484)
Supplement: S25 Fig — (PDF) [file pcbi.1006484.s025.pdf]

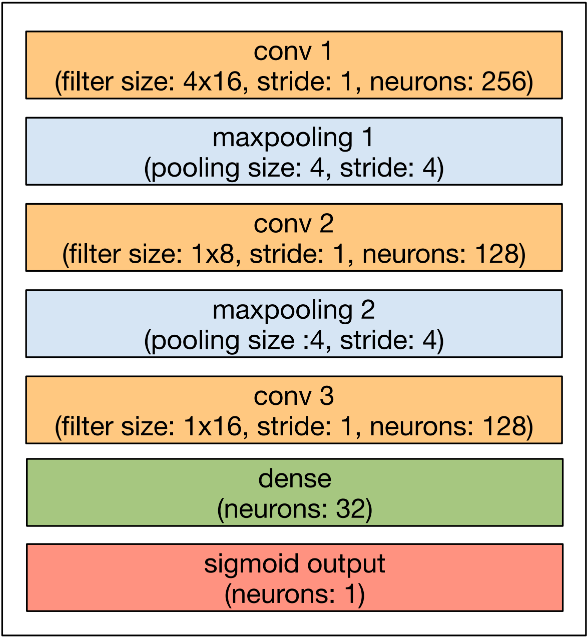
conv 1
(filter size: 4x16, stride: 1, neurons: 256)
maxpooling 1
(pooling size: 4, stride: 4)
conv 2
(filter size: 1x8, stride: 1, neurons: 128)
maxpooling 2
(pooling size :4, stride: 4)
conv 3
(filter size: 1x16, stride: 1, neurons: 128)
dense
(neurons: 32)
sigmoid output
(neurons: 1)
